# The pseudo-mitochondrial genome influences mistakes in heteroplasmy interpretation

**DOI:** 10.1186/1471-2164-7-185

**Published:** 2006-07-21

**Authors:** Ryan L Parr, Jennifer Maki, Brian Reguly, Gabriel D Dakubo, Andrea Aguirre, Roy Wittock, Kerry Robinson, John P Jakupciak, Robert E Thayer

**Affiliations:** 1 Genesis Genomics Inc, 1294 Balmoral Street, Thunder Bay, Ontario, P7B 5Z5, Canada; 2 Biochemical Science Division, National Institute of Standards and Technology, Gaithersburg, MD 20899, USA

## Abstract

**Background:**

Nuclear mitochondrial pseudogenes (numts) are a potential source of contamination during mitochondrial DNA PCR amplification. This possibility warrants careful experimental design and cautious interpretation of heteroplasmic results.

**Results:**

Here we report the cloning and sequencing of numts loci, amplified from human tissue and rho-zero (*ρ*^0^) cells (control) with primers known to amplify the mitochondrial genome. This paper is the first to fully sequence 46 paralogous nuclear DNA fragments that represent the entire mitochondrial genome. This is a surprisingly small number due primarily to the primer sets used in this study, because prior to this, BLAST searches have suggested that nuclear DNA harbors between 400 to 1,500 paralogous mitochondrial DNA fragments. Our results indicate that multiple numts were amplified simultaneously with the mitochondrial genome and increased the load of pseudogene signal in PCR reactions. Further, the entire mitochondrial genome was represented by multiple copies of paralogous nuclear sequences.

**Conclusion:**

These findings suggest that mitochondrial genome disease-associated biomarkers must be rigorously authenticated to preclude any affiliation with paralogous nuclear pseudogenes. Importantly, the common perception that mitochondrial template "swamps" numts loci precluding detectable amplification, depends on the region of the mitochondrial genome targeted by the PCR reaction and the number of pseudogene loci that may co-amplify. Cloning and relevant sequencing data will facilitate the correct interpretation. This is the first complete, wet-lab characterization of numts that represent the entire mitochondrial genome.

## Background

The unique maternal inheritance pattern of mitochondrial DNA (mtDNA), its small genome size, accelerated mutation rate, lack of recombination, and multiple copy number per cell, in comparison to nuclear DNA, are ideal biological traits for investigating evolution, population genetics and for forensic and medical applications. Thus, the mitochondrial genome has been used as a biosensor for the timing and movement of human populations in antiquity [1,2]. MtDNA analysis is routinely used in forensic biology to type biological material when degradation prevents nuclear STR amplification [3]. In addition, the entire mitochondrial molecule has potential medical utility because it can serve as a repository of cancer mutations and as a biosensor indicative of genetic alterations [4-13].

Frequently, identifying legitimate mtDNA mutations is confounded by heteroplasmy, a condition in which wild-type and mutant mitochondrial genomes co-exist in a cell. The interpretation of heteroplasmy can further be confounded by the widespread integration of portions of the mitochondrial genome into the nuclear genome [14,15]. These homologous, yet divergent nuclear and mtDNA sequences can be co-amplified in PCR reactions intended to replicate targeted mtDNA sequences only. Although this problem has previously been considered to be muted because of the high copy number of mtDNA over corresponding nuclear loci, caution is warranted [16]. For example, there are specific regions of the mitochondrial genome that have corresponding nuclear mitochondrial pseudogenes (numts) distributed across multiple chromosomes. Hence, there are regions of the mitochondrial genome that have a high nuclear copy number, which are not completely "swamped" during amplification. We report that some heteroplasmies detected in prostate cancer samples are a result of co-amplification of these multiple loci.

A large number of manuscripts addressing errors related to the interpretation of mtDNA and mtDNA heteroplasmy has been published [17-25]. Notably not all these errors are due to pseudogene co-amplification; however, mistakes from pseudogenes may increase with improved sequencing methods and highly sensitive re-sequencing microarray technologies that have a lower detection limit than traditional sequencing and which readily detect low-level heteroplasmy [11,26]. In some cases, if the heteroplasmy is inherited, it substantially increases the power of mutation detection, which becomes an important aspect since heteroplasmy has been reported as an early indication of disease [27-31]. In addition, if the disease process invokes mitochondrial depletion, this could increase nuclear pseudogene signal in reactions as a result of reduced mitochondrial genome copy number [32]. Loss of mitochondria has been described in several human cancers [33-36]. As well, the number of mitochondria and mtDNA copy number vary for different cell types [37-39]. These important matters relating to sequence interpretation have been generally neglected, in part, due to the lack of numt reference material, which would help investigators determine the relevance of detected mtDNA sequence variations. Hence, the need to validate somatic mitochondrial mutations is a pressing one.

Heteroplasmic issues have already complicated data obtained from other species. For example, in elephant hair, low mtDNA content is the reason why numts were co-amplified and misinterpreted as authentic mtDNA. In contrast, numts were not detected in DNA derived from elephant blood due to the presence of mitochondria-rich platelets [40]. Moreover, the hominid, *Gorilla*, is well known for significant numt interference with mitochondrial sequences, highlighting the need for diligence when interpreting human mtDNA heteroplasmy [41]. Not surprisingly, the effort for using mitochondrial cytochrome c oxidase as a primate "barcode" is plagued by numt amplification as well [42].

Further, laser capture microscopy has improved the ability to separate and analyze cancer cells, but because of the decreased amounts of sample DNA, many primer pairs are required to obtain a robust amplification of the entire mitochondrial genome [43]. Moreover, a sufficient number of cells must be captured to avoid incorporation errors associated with low template quantity [44]. This will also be relevant to studies that use formalin-fixed paraffin embedded samples [45]. The use of many primers means that smaller amplicons will be synthesized translating into a higher risk of co-amplification of numts and the potential misinterpretation of heteroplasmic calls.

There is limited *in silico *and wet-lab evidence indicating that fragments of the human mitochondrial genome are embedded in the nuclear DNA archive [46-50]. These findings emphasize the critical need to minimize erroneous interpretation of heteroplasmy, a vital necessity for precise forensic discrimination, evolutionary studies, and potential diagnostics. We provide evidence of numts for the entire mitochondrial genome by the amplification, cloning, and identification of numts from rho-zero (*ρ*^0^) cells and clinical cancer specimens. Here we present results from overlapping primers, which co-amplified nuclear embedded, paralogous mitochondrial sequence. Surprisingly, our data shows a relatively small number (when compared to hypothetical sequence information obtained from BLAST searches) of multiple nuclear loci that co-amplify with the mitochondrial genome. These findings demonstrate that accurate interpretation of heteroplasmy not only requires careful primer design and testing, but also indicates that a compendium of the sequence information from multiple-copy number numts is an important reference tool that will facilitate correct mtDNA interpretation and support reliable mitochondrial genome sequencing data.

## Results

### *ρ*^0 ^cells lack mtDNA

**Figure 1:**
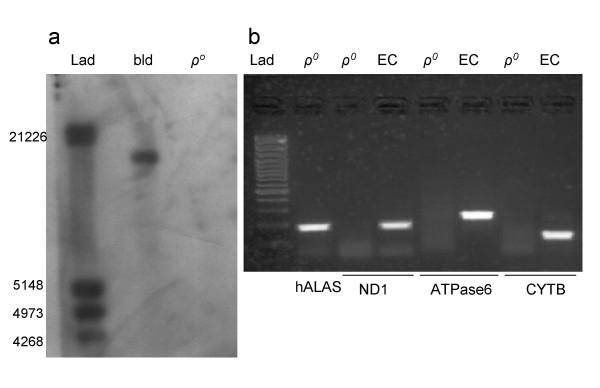
**ρ0 cells do not contain mtDNA.** a. Southern blot analysis of total DNA extracted from blood (bld) and ρ0 cells and probed with a full length mtDNA probe.  Note the absence of hybridization to ρ0 extracts. Lad is a DIG-labeled DNA molecular weight marker III (Roche).  b. PCR amplification of cDNA from ρ0 and epithelial cells (EC). Note the amplification of ρ0 cDNA with primers to the nuclear gene hALAS, whereas primers to ND1, ATPase6, and CYTB failed to amplify ρ0 cDNA, although they all amplified cDNA from EC.  Lad is a 100bp DNA size standard (Fermentas life sciences).

Rho-zero cells were evaluated for the presence of mtDNA. To ensure that total DNA extracted from *ρ*^0 ^cells were indeed devoid of mtDNA, we first performed Southern blot analyses on DNA extracts from *ρ*^0 ^cells. Blood was used as a positive control. No full length mitochondrial genome signal was observed in *ρ*^0 ^lanes when the blots were probed with mtDNA-specific probes (Figure 1a). We next performed PCR on the DNA extracts with primers specific to the mitochondrial coding regions. Again, there were no amplifications observed in the *ρ*^0 ^templates, while DNA isolated from blood was amplified, as expected (data not shown). We used RT-PCR to provide further evidence that *ρ*^0 ^cells are indeed devoid of mtDNA [51]. RT-PCR analysis was performed on RNA samples from *ρ*^0 ^cells and normal human skin tissue (epithelial cells) samples with primers to OXPHOS genes and a nuclear gene (positive control), 5-aminolevulinate synthase (hALAS) (Table 1). Whereas the hALAS primers amplified nuclear targets in template from *ρ*^0 ^and epithelial cells, there was no observable product with mtDNA primers for the *ρ*^0 ^cDNA template (Figure 1b), independently confirming the absence of mtDNA in these cells.

### Co-amplification of numts and mtDNA

**Figure 2:**
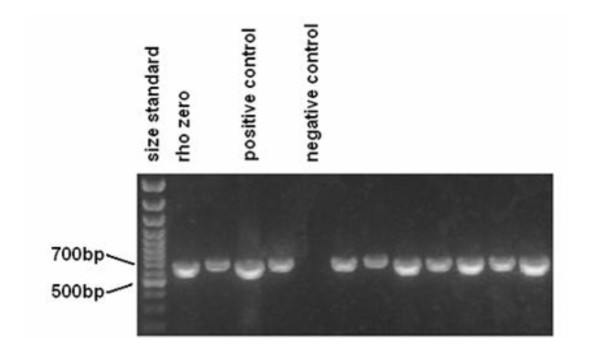
**Numts co-amplify from clinical samples.** A representative gel picture showing amplification of clinical samples with primers that also amplify ρ0 template is shown. Unlabelled lanes are the clinical samples.  Subsequent analysis of sequences from this amplification (see Figure 5) revealed the presence of pseudogene contamination.

**Figure 3:**
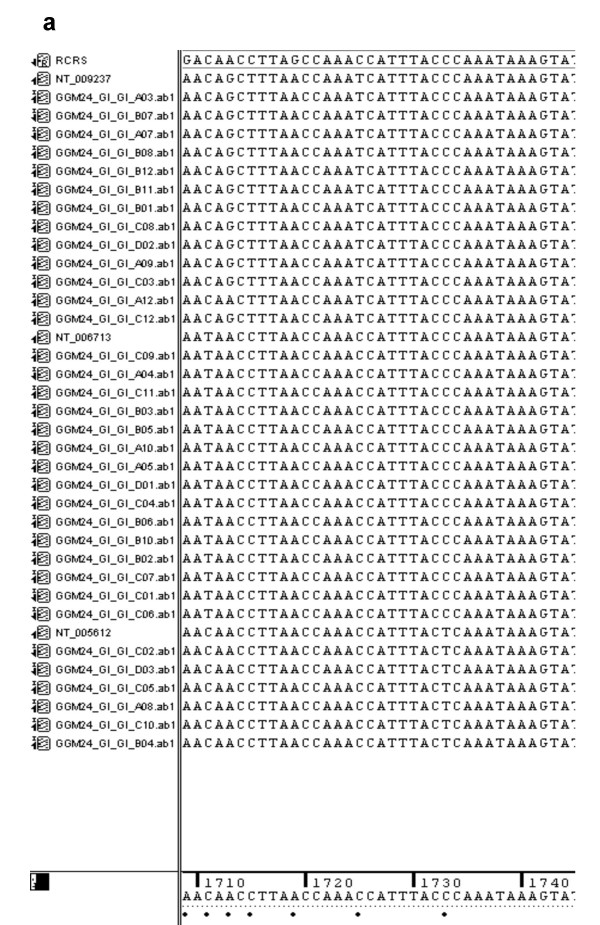
**Example of an alignment of three clones. **Example of an alignment of three clones (clones G C11. A1, G C3. A1 and G C5.C1) recovered from three chromosomes (Chr11 - NT_009237, Chr5 - NT_006713, and Chr3 - NT_005612) to the rCRS is shown.

**Figure 4:**
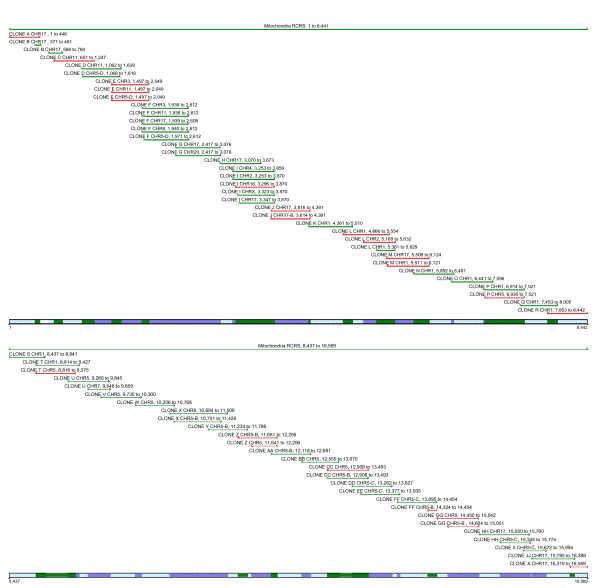
**The distribution of numt clones. **The distribution of numt clones (based on our primers) across the rCRS reveals regions of the rCRS with multiple numts copies. The pseudo-mitochondrial genome assembled from consensus numt sequences.The distribution of numt clones across the rCRS reveals sites that could be problematic when primers are designed to targets in these regions. Clone name, chromosomal location and rCRS positions are indicated above each clone**.**

Amplification of the complete mitochondrial genome was performed on human formalin fixed and paraffin embedded (FFPE) prostate cancer samples using a set of 34 primers (Table 2). Due to the amount and quality of DNA recovered, the average amplicon size was limited to 625 bp. Surprisingly, 24 (71%) of the primer sets co-amplified mitochondrial pseudogenes (Figure 2, and data not shown). A similar ratio was previously reported by an independent group using 38 primers (26/38 or 68%) [16]. In an effort to fully characterize numts that represent the entire mitochondrial genome, we redesigned the remaining 10 primers to co-amplify nuclear loci. Thus, we amplified template from *ρ*^0 ^cells and subsequently identified (via sequencing) the cloned fragments from the nucleus. A region of the D-loop (base pairs 16211-420 and 15-711) was recalcitrant to co-amplification using our mitochondrial primers. Therefore, two chromosome 17 specific primers were designed to capture this D-loop fragment (Table 2). Hence, a total of 36 primer sets were used to recover the entire mitochondrial genome from the nucleus. The sequences representative of the entire mitochondrial genome are provided as an additional material (additional file 1). Figure 3 is an example of an alignment to rCRS of three numt clones recovered using primer set 1488F/2084R (Table 2). These three clones were recovered form three separate chromosomes (Chr11 – NT_009237, Chr5 – NT_006713, and Chr3 – NT_005612). Similar alignment of our consensus cloned sequences enabled the assembly of a pseudo-mitochondrial genome (Figure 4).

**Figure 5:**
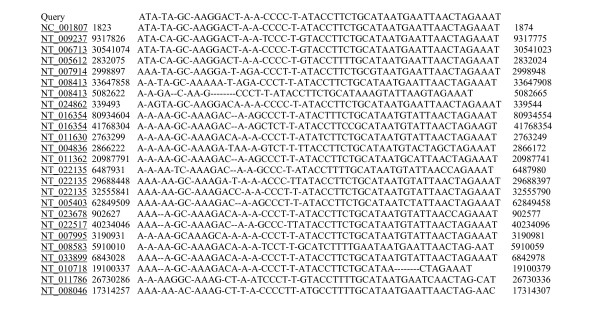
**Our primers recovered lower number of paralogous sequences compared to BLAST searches. **A BLAST search using the rCRS region covered by the three clones (Figure 3a)returns more numts representative of this region(25)than the three  obtained by our cloning data.

**Figure 6:**
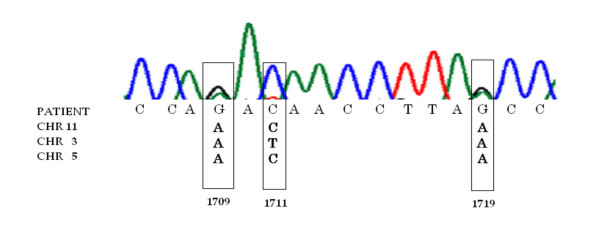
**A “piggyback” effect resulting from chromosomal copy number and shared divergent sites is demonstrated in a patient sample. **The chromatogram is from a patient for whom heteroplasmy at positions 1709 and 1719 were later noted tobe homologous to three chromosomes (3, 5, and 11), suggesting a possible co-amplification of numts in this instance.

The following chromosomes were represented in the data: 1, 2, 3, 4, 5, 7, 8, 9, 11, 16, 17, 20 and X. The number of paralogous sequences, in some instances, was lower than the number predicted from BLAST searches (Figure 3, 5). We demonstrate that there are only a limited number of multiple copy numts that potentially contribute to a heteroplasmic signal. Subsequently, we systematically inspected heteroplasmic sites observed in sequences from the prostate cancer samples for numt contribution using our cloned *ρ*^0 ^data as a reference. We discovered false heteroplasmic sites occurred when there was co-amplification of multiple numt loci with the same nucleotide at that particular site. Base pairs 1709, 1711 and 1719 in one specific amplicon (1488–2084, 16S rRNA) illustrate this point. The amplification of this specific region of the mitochondrial genome also co-amplified numts on chromosomes 3, 5 and 11 (Figures 3, 6). All three chromosomes have an A as opposed to a G, which correspond to mitochondrial positions 1709 and 1719. Using automated DNA sequencing, these multi-copy numts were detected as heteroplasmies at positions 1709 and 1719 (Figure 6). At position 1711, chromosomes 5 and 11 have a C as does the tissue; however, chromosome 3 has a T. A weak heteroplasmic signal is evident by a minute T peak, but because of the poor detection limit of fluorescent sequencing, this peak is virtually equivalent to background (Figure 6). Heteroplasmic signals were detected for other sites as well. For instance, the primer pair for the amplicon (3230–3893) co-amplified homologous numts on 5 different chromosomes (2, 4, 16, 17 and X). This region is evident in the pseudo-mitochondrial genome assembly from our clones (Figure 4).

### Multiple numt copies exist in the genome

**Figure 7:**
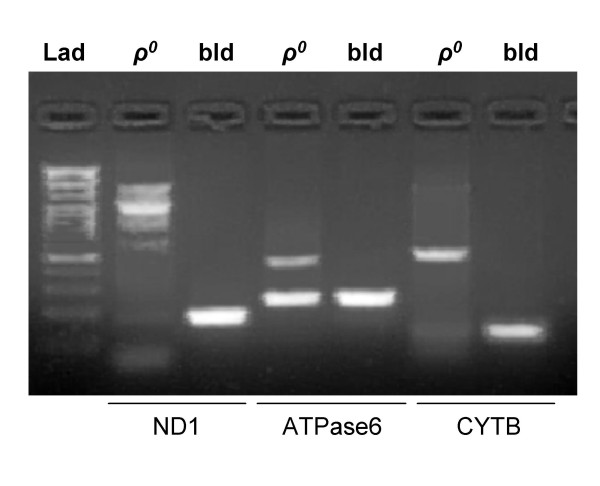
**Multiple numt copies are present in the nucleus.** PCR amplification of total DNA extracted from *?*^0^ and blood (bld) cells with primers targeting ND1, ATPase6 and CYTB genes. In contrast to the single amplicons obtained from blood, template from *?*^0^ contains additional high molecular weight amplicons.  Lad is a 100bp DNA size standard (Fermentas life sciences).

To cross-validate our cloned data, we analyzed genomic DNA from *ρ*^0 ^cells, blood, and human placenta using mitochondrial primers that co-amplified nuclear loci in the prostate cancer specimens. In blood and human placenta samples, a single mtDNA amplicon was consistently observed (Figure 7, and data not shown). Although sequence analysis of the prostate specimens detected numts, their signals were below the detection limit of agarose gel electrophoresis. In contrast, several primers consistently amplified numts from *ρ*^0 ^cells generating high molecular weight amplifications in addition to the expected mtDNA fragments (Figure 7). These findings confirm the presence of multiple numts loci in the genome and translate into real concern that numts are present in amplifications that produce more that one band or different size amplicons.

### Survey of mitochondrial genome mutations associated with disease suggests caution

Based on our findings that false heteroplasmic sites occurred when there was co-amplification of multiple numt loci with the same nucleotide at that particular site, we compared our cloning data to possible disease associated mutations listed on MITOMAP [52] and common sites were noted. In addition, a BLAST search was performed for these sites and hits held in common between the marker and cloning information were scored as well. Numerous commonalities were noted, which is cause for concern (Table 3).

## Discussion

In this study, we recovered and assembled the entire mitochondrial genome from nuclear loci. Moreover, this "pseudo-mitochondrial genome" involves numts from over half of the human complement of chromosomes, including the X chromosome. This suggests a widespread allocation of numts in the human nuclear genome. Surprisingly, this distribution was achieved with primers routinely used to amplify mtDNA, yet designed without consideration for numts. Seventy-one percent (24/34) of the primers co-amplified numts in prostate cancer tissue samples. This validates prior suggestions that numts are a potential source of misinformation and serves to illustrate the ease of co-amplification of both mtDNA and nuclear embedded paralogous mitochondrial DNA sequences [16]. Our data demonstrate that contrary to a consensus of opinions that the copy number of the mitochondrial genome "swamps" the signal from numts loci, there are circumstances which favor PCR recovery of numts, such as multiple pseudogene copy number [18]. For instance, heteroplasmic mutations had been associated with late-onset Alzheimer's disease [17,53]; however, these false heteroplasmies resulted from co-amplification of numts [19,54,55]. Indeed, human numts perplexed ancient DNA studies as well when it was reported that DNA had been recovered and amplified from a Cretaceous dinosaur bone [56]. This sequence corresponded to a human numt containing cytochrome *b *sequence [57], probably from reagent or sample contamination.

Direct pseudogene contribution is not always obvious and can confound suggested mtDNA biomarkers. For example, one set of primers in our data set amplifies tRNA^leu ^and ND1 (3230–3893). Subsequent cloning data identified co-amplification of paralogous numts on five chromosomes with this amplicon. Of specific interest are bases 3316, 3496, 3697 and 3796, which are reported as potential disease associated sites [52]. These sites are problematic since the reported base changes are consistent with pseudogene presentation in our data. Further, results from new sequencing technologies have suggested that homoplasmic signals may indeed be heteroplasmic in nature [11]. In addition, the heteroplasmic patterns seen at bps 3697 and 3796 are mirrored by the nuclear pseudogene patterns (Table 3).

Re-examination of the raw data from the above studies could address if the disease mutations are actually due to co-amplified numts. Potential markers must be thoroughly investigated to preclude the inclusion of false mutations in the interpretation of mtDNA mutations. BLAST searches of nuclear pseudogenes belie the possibility of widespread integration and/or replication of these sequences, since primers may amplify homologous numts embedded elsewhere in the genome. Thus, high copy numbers for these nuclear segments can produce potential misleading heteroplasmic signals.

Comparative marker studies require the same conditions and primers for meaningful results. For example, proposed MELAS sites T3250C and T3291C (tRNA^leu^) have paralogous nuclear sites [58-60]. Follow-up work by Akanuma *et al*. (2000) [61], demonstrate a corollary numt associated site for T3250C, but not T3291C. A BLAST search shows that these competing sequences are on separate chromosomes (17 and 20) indicating comparison of dissimilar data. In addition, the cloning data here identifies similar regions on chromosomes 2, 4 and 17 (Table 3). Clearly there are numerous paralogous loci for mitochondrial tRNA^leu^, the amplification of which depends on location and homology of primer sets.

Because of this association in our data, we compared our cloning results to the suggested mitochondrial genome disease associated sites listed on MITOMAP [52]. Results suggest that many mutations require meticulous scrutiny because of paralogous nuclear commonalities. Although many of these mutations may well be actual disease markers, the possibility of numt association may confound detection. For example, proposed prostate cancer mutations (**G**5913**A**, **G**5973**A **and **G**6081**A**) are identified, by cloning data, as resident on chromosomes 1 (2 homologous copies) and 17 even though the authors exercised precautionary measures by scanning a database of known nuclear pseudogenes [49,62]. A locus on chromosome 6 was identified as a potential co-amplification product, yet chromosomes 1 and 17 were not detected. Co-amplification of numts is primer dependent, which may explain the differences seen here; however, database limitations and the absence of extensive wet-lab numt data obscure the meaning of the marker. Particularly suspicious are those sites which demonstrate heteroplasmy in both normal and disease tissue. This may reflect a consistent pattern of numt amplification, an unintended characteristic of primer design. A subset of the marker work prior to the seminal numt work of Lopez *et al*. (1994) [14] may need clarification as well. For example, an ND2 lesion associated with Alzheimer's and Parkinson's diseases (**G**5460**T**/**A) **has modulated T and A nucleotides. Both mutations are seen in a BLAST search of numts. Moreover, our cloning data also identifies a G at this point, but not a T, again suggesting the relevance of primer selection; nevertheless, all possible modulations are described in this early work (see Kosel 1994. [63]), yet it remains a primary reference for this lesion.

If the use of mitochondrial DNA and in particular somatic mitochondrial genome mutations has important utility and medical merit, much of the data requires critical follow-up from a pseudogene perspective. Amplification of *ρ*^0 ^DNA template with primers to identify and eliminate those which co-amplify nuclear pseudogenes is a vital and necessary procedure [16]. For example, mitochondrial PCR protocols were simultaneously run on clinical samples and nucleic acid recovered from *ρ*^0 ^cells to identify and exclude co-amplification of numts in work by Coskun *et al*. [64]. Alternatively, data may be screened by amplification and sequencing of *ρ*^0 ^derived DNA and conflicting sites then backed out of actual data generated with identical primers; however, this approach is labor-intensive [43]. Phylogenetic analysis of the data would also help distinguish polymorphisms from authentic mutations [22]. In general, and unfortunately the advice by Parfait *et al*. has been largely ignored [16].

Our surprising results are not limited to short amplicons, but are also detected in much larger amplicons. For example, the overlapping amplification of chromosome 5 from bp 8816 to 15051 cautions against assuming that long amplicons are pseudogene free. These possibilities and characteristics of the nuclear genome must be considered when using mitochondrial sequence data for population, forensic or disease studies. Although designing and testing primers to avoid co-amplification of numts is a good laboratory practice, compilation of numts representative of the entire mitochondrial genome is valuable to catalog and characterize the overall nuclear burden of these sequences.

## Conclusion

Amplification of overlapping numts paralogous to the mitochondrial genome indicates that co-amplification of nuclear mitochondrial pseudogenes is a real problem for accurate sequence interpretation. Not only is co-amplification dependent on the particular amplicon used, but the copy number of these loci is also important. Only certain positions across the mitochondrial genome are associated with multiple copies of numts. Mitochondrial DNA heteroplasmy should be interpreted with caution since they can be the result of nuclear/cytoplasmic co-amplification. Herein, we have demonstrated the robust amplification of numts. This paper is the first to fully sequence the 46 paralogous DNA fragments that represent the entire mitochondrial genome using 36 primer pairs. This is a surprisingly low number, but reveals that only a limited number of paralogous numts are relevant when considering if heteroplasmic call are authentic mutations. Compilation of a complete data set of numt sequences will help others distinguish paralogous nuclear based heteroplasmy in forensic, population and medical applications.

## Methods

### Nucleic acid extraction

All research involving human tissue was approved by the Thunder Bay Regional Health Sciences Centre Ethics Committee in accordance with the Tri-Council Policy Statement for Research Involving Humans . Archived formalin fixed and paraffin embedded (FFPE) prostatectomy samples were laser capture microdissected (LCM) and DNA isolated by proteinase K digestion. DNA was isolated from blood using the UltraClean™ DNA BloodSpin kit (*MO BIO *laboratories, Inc). Human placenta DNA was purchased from Sigma-Aldrich (D4642). *ρ*^0^cells were prepared from a human osteocarcoma cell line 143B (ATCC CRL-8303) treated with ethidium bromide to deplete cytoplasmic mitochondrial DNA(kindly provided by Eric Shoubridge) [65]. Cells were grown to confluence in high glucose DMEM with pyruvate, L-glutamine, uridine (50 μg/ml) and 5% FBS. At confluence cultures were harvested and DNA was extracted using QIAmp DNA Mini Kit.

### Amplification, cloning and sequencing

Template from FFPE tissue samples and *ρ*^0 ^cells were amplified using 34 mitochondrial and 2 chromosome 17 primers. Using TaKaRa LA Taq DNA polymerase (Takara Bio Inc.), PCR reactions were performed using the following conditions: 1X LA PCR Buffer II (Mg^2+^plus), 0.4 mM each dNTP mixture, 1X BSA (New England Biolabs Inc.), 0.6 μM each primer, 1.25 Units LA Taq, 0.5% Ficoll 400 and 1 mM tartrazine (20,195-2, Aldrich). Total reaction volume was 25 μl. Cycling parameters were 94°C for 2 minutes, followed by 40 cycles of 94°C for 20 seconds, 30 seconds annealing at primer-specific optimized temperatures, and 72°C for 90 seconds. Cycling was performed on a DNA Engine Tetrad 2 (Bio-Rad, Hercules, CA). PCR products were purified, cloned and sequenced at Lark™ Technologies using in-house standard operating procedures (Houston, Texas). In general, 40 clones from each *ρ*^0 ^amplicon were selected and sequenced in both forward and reverse directions.

### Analysis

Sequences were analyzed using the Phred-Phrap-Consed software package [66]. The sequences were then grouped based on similarity and a megaBLAST search of NCBI database was performed (using default parameters) to identify all the nuclear co-ordinates of the fragments. This enabled the chromosomal location and nuclear copy number of each amplicon to be determined. Pairwise sequence alignment was performed between the revised Cambridge Reference Sequence (rCRS)[67] and the *ρ*^0 ^clones from the suite of amplicons covering the entire mitochondrial genome using the Sequencher™ software(Gene Codes Corporation).

### Southern Blotting

Mitochondrial genomes were cut with *Pvu*II from 2 ug of total DNA extracted from normal blood and *ρ*^0 ^cells. Digested product was electrophoresed through a 0.4% agarose gel and blotted onto a membrane (Hybond-N^+^, Roche Applied Sciences). Probes were generated from full length mtDNA (16.5 kb) by random primer labeling using the DIG System (Roche Diagnostics). Blots were incubated with probe, washed, blocked, incubated with anti-digoxigenin-AP fragments (Roche Applied Science) and reacted with a chemiluminescent substrate (CDP-Star^®^) and exposed to X-ray film (Kodak) as recommended by the DIG Application Manual for Filter Hybridization (Roche Diagnostics, 2000).

### PCR

For reverse transcriptase PCR analysis, total RNA was extracted from *ρ*^0 ^cells and a snap frozen skin sample using standard protocols outlined in the RNeasy Micro Kit manual (Qiagen). A DNase1 treatment step was included in the RNA extraction process to ensure the complete removal of all genomic DNA. We assessed RNA quantity and quality with the ND-1000 spectrophotometer (NanoDrop^® ^technologies) and by gel electrophoresis. First strand DNA was synthesized with the Omniscript^® ^RT (Qiagen) kit. 2 ul of the cDNA was amplified with primer sets to coding mitochondrial genes and a nuclear gene, 5-aminolaevulinate synthase (hALAS) (Table 3), using the PCR conditions described above except the annealing temperature for these primers was 54°C.

To examine for multiple copy numts, 50 ng of genomic DNA from *ρ*^0 ^cells, blood and human placenta were amplified as described above, using primer sets to OXPHOS genes,.

## Authors' contributions

RLP conceived of and supervised the study, and drafted the manuscript, JM, BR AA, and KR conducted experiments and participated in sequence alignment and data analysis, RW co-coordinated sample collection, GDD conducted experiments and helped draft the manuscript, JPJ provided intellectual insight and helped draft the manuscript, RET designed experiment and helped draft manuscript. All authors read and approved the final manuscript.

## Supplementary Material

Additional File 1 Sequences of numt clones. The chromosomal locations and sequences of numts amplified using our primers Click here for file

## Figures and Tables

**Table 1:** Sequences of four primer sets used in RT-PCR.

**Designation**	**Primer sequence (5'-3')**
hALASF	CCACTGGAAGAGCTGTGTGA
hALASR	ACCCTCCAACACAACCAAAG
ND1F	GAGCAGTAGCCCAAACAATC
ND1R	GGGTTCGGTTGGTCTCTGCTAG
ATP6F	CCATAAAATTATGAGCGGGCACAGTGATT
ATP6R	GGAAGGTTAATGGTTGATATTGCTAGG
CYTBF	CTAGCAACACTCCACCTCCTAT
CYTBR	GTAAGCCGAGGGCGTCTTTGCTTG

**Table 2:** 5' genome location (according to rCRS) and primer sequences for 36 primers including 34 (#3–36) used to amplify formalin fixed, paraffin embedded tissue. Primers in bold (1, 2) indicate chromosome (Chr) 17 specific primers redesigned to capture rest of D-loop fragment. The region spanned by 15971–16410 was split into two separate amplicons for the *ρ *^0 ^amplification (15673–16009 and 15777–16398).

		**Primer Sequence (5'-3')**	**Mitochondria Position**	**Chr**	**Accession Number**	**Nuclear Position**
**1**	**16319F**	**ATC GCT CAT GGT AGA TAG CAC**	16319-446	17	NT_024862.13	337395–338087
	**446R**	**TGT TAG TTG AGG GGT GAC TGTT**				
**2**	**374F**	**AAT CCA ACT CAA CCA GAG CCC A**	371–461	17	NT_024862.13	338011–338101
	**781R**	**TGA GCT GCA CTA ATG CGT GC**	*Nuclear Gap*	17	NT_024862.13	338102–338279
			574–783	17	NT_024862.13	338280–338489
3	615F	ATG TTT AGA CGG GCT CAC ATC ACC	651–1247	11	NT_009237.17	9318994-9318400
	1247R	CAA GAG GTG GTG AGG TTG ATC G				
4	1051F	ACA ATA GCT AAG ACC CAA ACT GGG AT	1062–1639	11	NT_009237.17	9318585-9318008
	1644R	CTC CTA AGT GTA AGT TGG GTG CTT TG	1068–1618	5	NT_006713.14	30541827-30541277
5	1488F	CGT CAC CCT CCT CAA GTA TAC TTC	1497–2049	11	NT_009237.17	9318150-9317598
	2084R	TAC AAG GGG ATT TAG AGG GTT CTG TG	1497–2049	5	NT_006713.14	30541398-30540846
			1497–2049	3	NT_005612.14	2832399-2832340
6	1938F	AGA GCA CAC CCG TCT ATG TAG CAA	1938–2612	11	NT_009237.17	9317709-9317035
	2612R	GGA ACA AGT GAT TAT GCT ACC TTT GCA C	1938–2612	3	NT_005612.14	2831958-2831284
			1939–2612	17	NT_024862.13	339611-340281
			1940–2612	8	NT_023678.15	902509-901844
			1971–2612	5	NT_006713.14	30540924-30540284
7	2417F	CAC TGT CAA CCC AAC ACA GGC AT	2402–3078	20	NT_011362.9	20987219-20986564
	3101R	TAG AAA CCG ACC TGG ATT ACT CCG	2417–3076	17	NT_024862.13	340090-340749
8	3021F	CGC TAT TAA AGG TTC GTT TGT TC	3070–3673	17	NT_024862.13	340743-341346
	3695R	ATC AGG GCG TAG TTT GAG TTT G				
9	3230F	GTT AAG ATG GCA GAG CCC GGT AA	3253–3859	4	NT_016354.17	80880160-80879559
	3893R	GTT CGG TTG GTC TCT GCT AGT GT	3253–3870	2	NT_005058.14	9588111-9587524
			3296–3870	16	NT_037887.4	3360917-3360343
			3323–3870	X	NT_011630.14	2761822-2761281
			3347–3870	17	NT_024862.13	341020-341543
10	3728F	CAT ATG AAG TCA CCC TAG CCA TC	3814–4391	17	NT_010718.15	19103293-19103865
	4417R	TTT AGC TGA CCT TAC TTT AGG ATG GG	3816–4391	17	NT_024862.13	341489-342064
11	4337F	ATG AGA ATC GAA CCC ATC CCT GAG	4361–5010	1	NT_077913.2	43543-44192
	5035R	CAT CCT ATG TGG GTA ATT GAG GAG T				
12	4867F	GAC AAA AAC TAG CCC CCA TCT CAA	4866–5554	1	NT_077913.2	44048–44736
	5646R	GCT TAA TTA AAG TGG CTG ATT TGC GT	5169–5632	2	NT_005403.15	62851661-62852120
13	5468F	CAC GCT ACT CCT ACC TAT CTC	5508–6124	17	NT_024862.13	343174–343794
	6145R	CAG TTG CCA AAG CCT CCG ATT ATG	5511–6121	1	NT_077913.2	44693–45303
14	5867F	CAA TGC TTC ACT CAG CCA TTT TAC C	5892–6481	1	NT_077913.2	45074–45663
	6482R	GAC TGC TGT GAT TAG GAC GG				
15	6418F	AAC CCC CTG CCA TAA CCC AAT AC	6441–7056	1	NT_077913.2	45623-46239
	7082R	GAA GCC TCC TAT GAT GGC AAA TAC AG				
16	6911F	TGC AGT GCT CTG AGC CCT AGG ATT	6914–7521	1	NT_077913.2	46097-46704
	7554R	CTT TGA CAA AGT TAT GAA ATG GTT TTT CTA ATA	6935–7521	5	NT_034772.5	1804907-1804321
17	7400F	CCC ACC CTA CCA CAC ATT CGA A	7453–8005	1	NT_077913.2	46636–47188
	8029R	GGC TTC AAT CGG GAG TAC TAC TCG				
18	7829F	CGC ATC CTT TAC ATA ACA GAC GAG G	7853–8472	1	NT_077913.2	47036–47622
	8441R	GTT GGG TGA TGA GGA ATA GTG TAA GG				
17	8346F	CAACACCTCTTTACAGTGAAATGCCC	8471–8941	1	NT_077913.2	47652–48122
	8959R	CGATAATAACTAGTATGGGGAT				
20	8814F	CCA ACT ATC TAT AAA CCT AGC C	8814–9427	1	NT_077913.2	47995–48594
	9413R	GCC TTG GTA TGT GCT TTC TCG TGT	8816–9375	5	NT_034772.5	1803025-1802466
21	9247F	GCC CAT GAC CCC TAA CAG G	9268–9845	5	NT_034772.5	1802573-1801994
	9868R	CGG ATG AAG CAG ATA GTG AGG	9545–9859	7	NT_079592.1	56744603-56744909
22	9711F	CTG GGT CTC TAT TTT ACC CTC C	9730–10300	5	NT_034772.5	1802112-1801539
	10285R	GGT AGG GGT AAA AGG AGG GCA				
23	10198F	CCC GCG TCC CTT TCT CCA T	10206–10768	5	NT_034772.5	1801633-1801071
	10766R	TTA GCA TTG GAG TAG GTT TAG G				
24	10696F	CCC TAC TAG TCT CAA TCT CCA A	10694–11509	9	NT_008470.17	2194293–2194999
	11426R	CTT CGA CAT GGG CTT TAG GGA G	10751–11426	5	NT_034772.5	36678748-36678073
25	11210F	TTC TAC ACC CTA GTA GGC TCC CTT	11234–11786	5	NT_034772.5	36678265-36677713
	11813R	GTA GAG TTT GAA GTC CTT GAG AGA GG				
26	11629F	AAT CAG CCA CAT AGC CCT CGT AG	11651–12205	5	NT_034772.5	36677848-36677294
	12231R	GTT AGC AGT TCT TGT GAG CTT TCT CG	11841–12206	5	NT_034772.5	1799998-1799633
27	12096F	TCC TAT CCC TCA ACC CCG ACA T	12118–12681	5	NT_034772.5	36677381-36676818
	12709R	GGA AGA TGA GTA GAT ATT TGA AGA ACT G				
28	12528F	GAA CTG ACA CTG AGC CAC AAC C	12550–13070	5	NT_034772.5	1799289-1798769
	13096R	CAA CTA TAG TGC TTG AGT GGA GTA GG				
29	12882F	CAT CCT CGC CTT AGC ATG ATT TAT CC	12908–13493	5	NT_034772.5	36676591-36676006
	13516R	GGT CTT TGG AGT AGA AAC CTG TG	12908–13493	5	NT_034772.5	1798931-1798346
30	13239F	CGT AGC CTT CTC CAC TTC AAG TC	13262–13827	5	NT_023148.12	2217633-2218198
	13851R	GTT GAG GTC TAG GGC TGT TAG AAG				
31	13354F	TTT ATG TGC TCC GGG TCC ATC AT	13377–13935	5	NT_023148.12	2217748-2218306
	13957R	CTA GAT AGG GGA TTG TGC GGT G				
32	13838F	CCC TAG ACC TCA ACT ACC TAA CC	13895–14454	5	NT_023148.12	2218266-2218823
	14458R	GAT GGC TAT TGA GGA GTA TCC T	14324–14535	5	NT_034772.5	36675175-36674964
33	14339F	ACC CCA TCA TAC TCT TTC ACC C	14550–15042	5	NT_034772.5	1797288-1796796
	15052R	TAG GCC TCG CCC GAT GTG TA	14604–15051	5	NT_034772.5	36674895-36674448
34	14971F	TGG CTG AAT CAT CCG CTA CCT T	15041–15785	17	NT_024862.13	336116-336858
	15786R	AAA GGG TAG CTT ACT GGT TGT CC	15345–15774	5	NT_023148.12	2219715-2220144
35	15673F	ATC CAA ACA ACA AAG CAT AAT ATT TC	15672–15994	5	NT_023148.12	2220042-2220364
	16009R	AAT TAG AAT CTT AGC TTT GGG TG				
36	15777F	GCT ACC CCT TCA TCA CCT TC	15785–16388	17	NT_024862.13	336858-337463
	16398R	CAA CGG ACC ACT ATC TGA GGG				

**Table 3:** 

**Locus**	**Disease**	**Allele**	**Hom/Het**	**BLAST hits**	**Cloning data**
Leu	MM/CPEO	**T**3250**C**	-/+	C = 16; A = 1	C = 17
Leu	CPEO	**C**3254**T**	+/-	T = 18; A = 2	T = 2, 4 and 17
Leu	MELAS	**T**3291**C**	-/+	C = 17	C = 2, 4 and 17
ND1	NIDDM; LHON; PEO	**G**3316**A**	+/-	A = 17	A = 2, 4, 16, 17
ND1	LHON	**G**3496**T**	+/-	T = 14; A = 2	T = X, 2, 4, 16, & 17
ND1	MELAS	**G**3697**A**	-/+	A = 3; C = 1	A = 2 & 4; G = X, 16, 17
ND1	Adult-Onset Dystonia	**A**3796**G**	-/+	G = 6	A = 2,4 & 17; G = X & 16
	ADPD/Hearing Loss &				
Gln	Migrane	**T**4336**C**	+/+	C = 1	C = 17
ND2	AD;PD	**G**5460**T**/**A**	+/+	T = 5; A = 9	G = 1 (2); T = 2
Asn	CPEO/MM	**G**5703**A**	-/+	A = 24	A = 17
COI	PCA	**G**5913**A**	+/-	A = 18; T = 1	G = 1(2); A = 17
	Myogloinuria; Exercise				
CO1	Intolerance	**G**5920**A**	-/+	A = 0	G = 1 (2); A = 17
CO1	PCA	**G**5973**A**	+/-	A = 3	G = 1(2); A = 17
COI	PCA	**G**6081**A**	+/-	A = 1; T = 1	G = 1 (2); A = 17
Ser	MM/Exercise Intolerance	**G**7497**A**	+/+	A = 14	A = 5
ATP6	LDYT	**G**8950**A**	+/-	A = 2	A = 5
ATP6	Leigh Disease	**T**9185**C**	-/+	C = 1	C = 5
ND4	MELAS	**A**11084**G**	+/+	G = 9	G = 9
His	MICM	**G**12192**A**	+/-	A = 9	G = 5 (3); A = 2(5)
ND5	LHON	**G**13708**A**	+/-	A = 15	A = 5 & 9
	Paracrystalline Inclusions				
CYTB	with Exercise Intolerance	**G**15497**A**	+/-	A = 2	A = 17
Thr	Multiple Sclerosis	**G**15927**A**	+/-	A = 5	G = 5; A = 17
	Type 2 Diabetes;				
D-Loop	Cardiomyopathy	**T**16189**C**	+/+	C = 0	C = 17

**Hom: **homoplasmy; **Het: **heteroplasmy;**BLAST hits: **number of times a unique numt nucleotide location was returned with a BLAST search; **Cloning data: **designates chromosome(s) location of nucleotide from cloning data; **AD: **Alzeimer's Disease; **ADPD: **Alzeimer's and Parkinson's Disease; **PD: **Parkinson's Disease; **CPEO: **Chronic Progressive External Opthalmoplegia; **PEO: **Progressive External Opthalmoplegia; **LDYT: **Leber's hereditary optic neuropathy and Dystonia; **MELAS: **Mitochondrial Encephalomyopathy, Lactic Acidosis and Stroke-like episodes; **LHON: **Leber's Hereditary Optic Neuropathy; **MICM: **Maternally Inherited Cardiomyopathy; **MM: **Mitochondrial Myopathy;**NIDDM: **Non-Insulin Dependent Diabetes; **PCA: **Prostate Cancer.
