# Supplementary material for: The pseudo-mitochondrial genome influences mistakes in heteroplasmy interpretation
Source: BMC Genomics. 2006 Jul 21;7:185. doi: 10.1186/1471-2164-7-185 (PMC1538596; doi:10.1186/1471-2164-7-185)
Supplement: Additional File 1 — Sequences of numt clones. The chromosomal locations and sequences of numts amplified using our primers [file 1471-2164-7-185-S1.pdf]

>CHR 1 NT_077913.2 46636-47188
GGAAGGAATCGAACCCCCCAAAGCTGGTTTCAAGCCAACCCCATGGCCTCCATGAC
TTTTTCAAAAAGATATTAGAAAAACCATTTCATAACTTTGTCAAAGTTAAATTATAG
GCTAAATCCTATATATCTTAATGGCACATGCAGCGCAAGTAGGTCTACAAGACGCTA
CTTCCCCTATCATAGAAGAGCTTATCATCTTTCATGATCACGCCCTCATAATCATTTT
CCTTATCTGCTTCCTAGTCCTGTACGCCCTTTTCCTAACACTCACAACAAAACTAACT
AATACTAACATCTCAGACGCTCAGGAAATAGAAACCGTCTGAACTATCCTGCCCGCC
ATCATCCTAGTCCTTATCGCCCTCCCATCCCTACGCATCCTTTACATAACAGACGAG
GTCAACGATCCCTCCTTTACCATCAAATCAATTGGCCATCAATGGTACTGAACCTAC
GAATACACCGACTACGGCGGACTAATCTTCAACTCCTACATACTTCCCCCATTATTC
CTAGAACCAGGCGACCTGCGACTCCTTGACGTTGACAAT

>CHR 1 NT_077913.2 47036-47595
GTCAACGATCCCTCCTTTACCATCAAATCAATTGGCCATCAATGGTACTGAACCTAC
GAATACACCGACTACGGCGGACTAATCTTCAACTCCTACATACTTCCCCCATTATTC
CTAGAACCAGGCGACCTGCGACTCCTTGACGTTGACAATCGAGTAGTACTCCCGGTT
GAAGCCCCCATTCGTATAATAATTACATCACAAGACGTCTTACACTCATGAGCTGTC
CCCACATTAGGCTTAAAAACAGATGCAATTCCCGGACGTCTAAACCAAACCACTTTC
ACTGCTACACGACCAGGGGTATACTACGGCCAATGCTCTGAAATCTGTGGAGCAAA
CCAGTTTTATGCCCATCGTCCTAGAATTAATTCCCCTAAAAATCTTTGAAATAGGGC
CCGTATTTACCCTATAGCACCCCCTCTACCCCCTCTAGAGCCCACTGTAAAGCTAAC
TTAGCATTAACCTTTTAAGTTAAAGATTAAGAGAACCAACACCTCTTTACAGTGAAA
TGCCCCAACTAAATACTACCGTATGACCCACCATAATTACCCCCATACTCCTTACAC
TATTCCTCATCACCCAACA

>CHR 1 NT_077913.2 47617-48122
CCAACTAAAAATATTAAATACAAATTACCACCTACCTCCCTCACCAAAGCCCATAAA
AATAAAAAACTATAACAAACCCTGAGAACCAAAATGAACGAAAATCTGTTCACTTC
ATTCATTGCCCCCACAATCCTAGGCCTACCCGCCGCAGTACTGATCATTCTATTTCCC
CCTCTATTGATCCCCACCTCCAAATATCTCATCAACAACCGACTAATTACCACCCAA
CAATGACTAATCCAACTAACCTCAAAACAAATGATAGCCATACACAACACTAAGGG
ACGAACCTGATCTCTTATACTAGTATCCTTAATCATTTTTATTGCCACAACTAACCTC
CTCGGACTCCTGCCTCACTCATTTACACCAACCACCCAACTATCTATAAACCTAGCC
ATGGCCATCCCCTTATGAGCGGGCGCAGTGATTATAGGCTTTCGCTCTAAGATTAAA
AATGCCCTAGCCCACTTCTTACCACAAGGCACACCTACACCCCTTATCC

>CHR 1 NT_077913.2 47995-48594
CCAACTATCTATAAACCTAGCCATGGCCATCCCCTTATGAGCGGGCGCAGTGATTAT
AGGCTTTCGCTCTAAGATTAAAAATGCCCTAGCCCACTTCTTACCACAAGGCACACC
TACACCCCTTATCCCTATACTAGTTATTATCGAAACCATCAGCCTACTCATTCAACCA
ATAGCCCTGGCCGTACGCCTAACCGCTAACATTACTGCAGGCCACCTACTCATGCAC
CTAATTGGAAGCGCCACACTAGCAATATCAACTATTAACCTTCCCTCTACACTTATC
ATCTTCACAATTCTAATTCTACTGACTATCCTAGAAATCGCTGTCGCCTTAATCCAAG
CCTACGTTTTTACACTTCTAGTAAGCCTCTACCTGCACGACAACACATAATGACCCA
CCAATCACATGCCTATCATATAGTAAAACCCAGCCCATGGCCCCTAACAGGGGCCCT
CTCAGCCCTCCTAATGACCTCCGGCCTAGCCATGTGATTTCACTTCCACTCCACAACC

CTCCTCATACTAGGCCTACTAACCAACACACTAACCATATACCAATGATGGCGCGAT
GTAACACGAGAAAGCACATACCAAGGCAAGCCGAATTCCAGC

>CHR 1 NT_077913.2 43543-44192
AATCCAAAATTCTCCGTGCCACCTATCACACCCCATCCTAAAGTAAGGTCAGCTAAA
TAAGCTATCGGGCCCATACCCCGAAAATGTTGGTTATATCCTTCCCGTACTAATTAA
TCCCCTGGCCCAACCCGTCATCTACTCTACCATCTTTGCAGGCACACTCATCACAGC
GCTAAGCTCGCACTGATTTTTTACCTGAGTAGGCCTAGAAATAAACATGCTAGCTTT
TATTCCAGTTCTAACCAAAAAAATAAACCCTCGTTCCACAGAAGCTGCCATCAAGTA
TTTCCTCACGCAAGCAACCGCATCCATAATCCTTCTAATAGCTATCCTCTTCAACAAT
ATACTCTCCGGACAATGAACCATAACCAATACCACCAATCAATACTCATCATTAATA
ATCATAATGGCTATAGCAATAAAACTAGGAATAGCCCCCTTTCACTTCTGAGTCCCA
GAGGTTACCCAAGGCACCCCTCTGACATCCGGCCTGCTCCTTCTCACATGACAAAAA
CTAGCCCCCATCTCAATCATATACCAAATTTCTCCCTCATTAAACGTAAGCCTTCTCC
TCACTCTTTCAATCTTATCCATCATGGCAGGCAGTTGAGGTGGATTAAACCAAACCC
AACTACGCAAAATCTTAGCAT

>CHR 1 NT_077913.2 44048-44736
TGACAAAAACTAGCCCCCATCTCAATCATATACCAAATTTCTCCCTCATTAAACGTA
AGCCTTCTCCTCACTCTTTCAATCTTATCCGTCATGGCAGGCAGTTGAGGTGGATTAA
ACCAAACCCAACTACGCAAAATCTTAGCATACTCCTCAATTACCCACATAGGATGAA
TAACAGCAGTTCTACCGTACAACCCTAACATAACCATTCTTAATTTAACTATTTATAT
TATCCTAACTACTACCGCATTCCTACTACTCAACTTAAACTCCAGCACCACAACCCT
ACTACTATCTCGCACCTGAAACAAGCTAACATGACTAACACCCTTAATTCCATCCAC
CCTCCTCTCCCTAGGAGGCCTGCCCCCGCTAACCGGCTTTTTGCCCAAATGGGCCAT
TATCGAAGAATTCACAAAAAACAATAGCCTCATCATCCCCACCATCATAGCCATCAT
CACCCTCCTTAACCTCTACTTCTACCTGCGCCTAATCTACTCCACCTCAATCACACTA
CTCCCTATATCTAACAACGTAAAAATAAAATGACAGTTTGAACACACAAAACCCAC
CCCATTCCTCCCCACACTCATCGCCCTTACCACACTGCTCCTACCTATCTCCCCTTTT
ATGCTAATAATCTTATAGAAATTTAGGTTAAATACAGACCAAGAGCCTTCAAAGCCC
TCAGTAAGTTGCAATACTTAATTTCTGCAACAGCTAAGGACTGCAAAACCCCACTCT
GCATCAACTGAACGCAAATC

>CHR 1 NT_077913.2 44693-45303
TAGAAATTTAGGTTAAATACAGACCAAGAGCCTTCAAAGCCCTCAGTAAGTTGCAAT
ACTTAATTTCTGCAACAGCTAAGGACTGCAAAACCCCACTCTGCATCAACTGAACGC
AAATCAGCCACTTTAATTAAGCTAAGCCCTTACTAGACCAATGGGACTTAAACCCAC
AAACACTTAGTTAACAGCTAAGCACCCTAATCAACTGGCTTCAATCTACTTCTCCCG
CCGCCGGGAAAAAAGGCGGGAGAAGCCCCGGCAGGTTTGAAGCTGCTTCTTCGAAT
TTGCAATTCAATATGAAAATCACCTCAGAGCTGGTAAAAAGAGGCTTAACCCCTGTC
TTTAGATTTACAGTCCAATGCTTCACTCAGCCATTTTACCTCACCCCCACTGATGTTC
GCCGACCGTTGACTATTCTCTACAAACCACAAAGACATTGGAACACTATACCTATTA
TTCGGCGCATGAGCTGGAGTCCTAGGCACAGCTCTAAGCCTCCTTATTCGAGCCGAA
CTGGGCCAGCCAGGCAACCTTCTAGGTAACGACCACATCTACAACGTTATCGTCACA
GCCCATGCATTTGTAATAATCTTCTTCATAGTAATACCCA

>CHR 1 NT_077913.2 45074-45663
TCACCCCCACTGATGTTCGCCGACCGTTGACTATTCTCTACAAACCACAAAGACATT
GGAACACTATACCTATTATTCGGCGCATGAGCTGGAGTCCTAGGCACAGCTCTAAGC
CTCCTTATTCGAGCCGAACTGGGCCAGCCAGGCAACCTTCTAGGTAACGACCACATC
TACAACGTTATCGTCACAGCCCATGCATTTGTAATAATCTTCTTCATAGTAATACCCA
TCATAATCGGAGGCTTTGGCAACTGACTAGTTCCCCTAATAATCGGTGCCCCCGATA
TGGCGTTTCCCCGCATAAACAACATAAGCTTCTGACTCTTACCCCCCTCTCTCCTACT
CCTGCTTGCATCTGCTATAGTGGAGGCCGGCGCAGGAACAGGTTGAACAGTCTACCC
TCCCTTGGCAGGGAACTACTCCCACCCTGGAGCCTCCGTAGACCTAACCATCTTCTC
CTTACACCTAGCAGGTATCTCCTCTATCTTAGGAGCCATCAATTTCATCACAACAATT
ATTAATATAAAACCCCCTGCCATAACCCAATACCAAACGCCCCTTTTCGTCTGATCC
GTCCTAATCACAGCAGT

>CHR 1 NT_077913.2 45623-46239
CAAACGCCCCTTTTCGTCTGATCCGTCCTAATCACAGCAGTCTTACTTCTCCTATCTC
TCCCAGTCCTAGCCGCTGGCATCACTATACTACTAACAGACCGTAACCTCAACACCA
CCTTCTTCGACCCAGCCGGAGGAGGAGACCCCATTCTATACCAACACCTATTCTGAT
TTTTCGGTCACCCTGAAGTTTATATTCTCATCCTACCAGGCTTCGGAATAATCTCCCA
TATTGTAACTTACTACTCCGGAAAAAAAGAACCATTTGGATACATAGGTATGGTCTG
AGCTATGATATCAATTGGCTTCCTAGGGTTTATCGTGTGAGCACACCATATATTTAC
AGTAGGAATAGACGTAGACACACGAGCATATTTCACCTCCGCTACCATAATCATCGC
TATCCCCACCGGCGTCAAAGTATTTAGCTGACTCGCCACACTCCACGGAAGCAATAT
GAAATGATCTGCTGCAGTGCTCTGAGCCCTAGGATTTATTTTTCTTTTCACCGTAGGT
GGCCTGACTGGCATTGTATTAGCAAACTCATCACTAGACATCGTACTACACGACACG
TACTACGTTGTAGCCCACTTCCACTATGTCCTATCAATAGGAG

>CHR 1 NT_077913.2 46097-46704
AGTGCTCTGAGCCCTAGGATTTATTTTTCTTTTCACCGTAGGTGGCCTGACTGGCATT
GTATTAGCAAACTCATCACTAGACATCGTACTACACGACACGTACTACGTTGTAGCC
CACTTCCACTATGTCCTATCAATAGGAGCTGTATTTGCCATCATAGGAGGCTTCATTC
ACTGATTTCCCCTATTCTCAGGCTACACCCTAGACCAAACCTACGCCAAAATCCATT
TCGCTATCATATTCATCGGCGTAAATCTAACTTTCTTCCCACAACACTTTCTCGGCCT
ATCCGGAATGCCCCGACGTTACTCGGACTATCCCGATGCATACACCACATGAAATAT
CCTATCATCTGTAGGCTCATTCATTTCTCTAACAGCAGTAATATTAATAATTTTCATA
ATTTGAGAAGCCTTCGCTTCGAAGCGAAAAGTCCTAATAGTAGAAGAACCCTCCATA
AACCTGGAGTGACTATATGGATGCCCCCCACCCTACCACACATTCGAAGAACCCGTA
TACATAAAATCTAGACAAAAAAGGAAGGAATCGAACCCCCCAAAGCTGGTTTCAAG
CCAACCCCATGGCCTCCATGACTTTTTCAAAAAGA

>CHR 2 NT_005058.14 9588111-9587524
TTGCATAAAACTTAAAACTTTATAATCTGAGGTTCAACTCCTCTTAACATACCTATAA
TTAACCTTCTCCTACTTATTGTCCCCACTCTAATCGCTATAGCATTCCTTACACTCTTT
GAATGAAAAATCTTAGGCTATATACAACTACGCAAAGGACATAACATCATGGGCCC
CTATGGACTGCTCCACCATTCGCTGATGCAATAAAACTTTTTACCAAAGAACCATTA
TGGCCCTCAACATCTAATATTACCCTCTATATTATTGCTCCAACCCTGGCCCTCTCCT
TTTGTGAACCCCCCTGCCTATACCAGATCCCCTAATTTAGGCCTATTTATATTAGCCA

CATCAAGCTTAGCTGTCTATTCTATTTTATGGTCAGAATGAGCATCCAATTCAAATTA
TGCACTAATAGGCACATTACGAGCTGTAGCCCAGACAATTTCATATGAGGTCACCCT
AGCCATTATTCTGTTATCAGTTCTACTAATAAGTGGCTCACTTAACTTGTACAAACTC
ATCACAACACAAGAATTTCTCTGACTGCTCCTATCATCATGGCCCCTAGCCATAATA
TGATTTACCTCT

>CHR 2 NT_005403.15 62851661-62852120
TGAAAGAAATTAGCATGGTTGACACCTATAATTCCACTAATTCCACTATCCCTACGA
GGTTTACCCCCATTACAGGGTTCCTGCCTAAATGAATCATCATCCAAGGATTTACAA
AAAATTATAGCCTTGGCACCCCAACCATTATAGCTATCATAACCCTACTCAACCTGT
ACTCTTACATACACCTCATTTATTCCATCTTGGTAACACTATTCCCCACATCTAATAA
TATGAAACTAAAATGACAATTCGAAATCGCAAAACCCACACTACTCCCTCCTCACTT
GTCATCTCTTCTACCGTCCTCTTACCCACCTCTCCATTAATACTAACTATAACTTAGA
AATTTAGTTTAAATAAGACCAAGGGCCTTCAAAGCCCTTAGTAAGTAAATGACACTT
AATTTCTGTAACAGACCTAAGGGCTGCAAGACTCTATTCTGCATCAGTTGAACGCAA
ATCAGC

>CHR 3 NT_005612.14 2831958-2831284
AGAGCACACCCGTCTATGTAGCAAAATAGTGGGAAGATTCGTAGGTAGAGGCGGCA
AACCTACCGAGTCTGGTGATAGCTGGTTGTCCAAGACAGAATCTTAGTTCAACTTTA
AATTTACCCACAGAACCCTCCAAATCCCCCTGTAAATTTAACTGTTAGTCCAAAGAG
GAACAGCTCTTTGGACACTAGGAAAAAACCTTGTAAAGAGAGTAAAAAATTTAATA
CCCATAGTAGGCCTAAAAGCAGCCACCAATTAAGAAAGCGTTCAAGCTCAACACCC
ATCGTCTAAAAAATCCCAAACATACAACTGAGCTCCTTGCACTCAATTGGACCAATC
TATTACCTTATAGAAGAACTAATGTTAGTATAAGTAACATGAAAACATTCTCCTCCG
CATAAGCCTACATCAGACCAAAATATTAAACTGACAATTAACAGCCCAATATCTACA
ATCAACCAACAAGCCATTATTACCCTCACTGTTAACCCAACACAGGCATGCCCACAA
GGAAAGGTTAAAAAAAGTAAAAGGAACTCGGCAAATCTTACCCCGCCTGTTTACCA
AAAACATCACCTCTAGCATTATCAGTATTAGAGGCACCGCCTGCCCGGTGACATATG
TTTAACGGCCGCGGTACCCTAACCGTGCAAAGGTAGCATAATCACTTGTTCC

>CHR 3 NT_005612.14 2832399-2832340
CCTCAAGTATACTTCAAAGGATATTTAACTAAAACCCCTACGCATTTATATAGAGGA
AACAAGTCGTAACATGGTAAGTGTAGTGGAAAGTGCACTTGGACGAACCAGAGTGT
AGCTTAACATAAAGCACCCAACTTACACTTAGGAGATTTCAACTCAACTTGACCACT
CTGAGCCAAACCTAGCCCTAAACCCGTTCCACCTTACTACCAAATAACCTTAACCAA
ACCATTTACCCAAATAAAGTATAGGCGATAGAAATTGTAAACCGGCGCAATAGATA
TAGTACCGCAAGGGAAAGATGAAAAATTATAACCAAGCATAATACAGCAAGGACTA
ACCCCTGTACCTTTTGCATAATGAATTAACTAGAAATAACTTTGCAAAGAGAACCAA
AGCTAAGGCCCCCGAAACCAGACGAGCTACCTAAGAACAGCTAAAAGAGCACACCC
GTCTATGTAGCAAAATAGTGGGAAGATTTATAGGTAGAGGCGACAAACCTATCGAG
CCTGGTGATAGCTGGTTGTCCAAGATAGAATCTTAGTTCAACTTT

>CHR 4 NT_016354.17 80880160-80879559
TTGCATAAAACTTAAAATTTTATAATCAGAGGTTCAACTCCTCTTCTTAACAATATGG
CTATAATTAACCTTCTCCTACTTATTATCCCCACTCTTATTGCTATAGCATTCCTTACA

CTCATCGAATGAAAAATCTTAGGCTATACACAACTATGCAAAGGACCCAAAATTGT
AGGTCCCTATGGGCTGCTTCAGCCATTCGCTGATGGAATAAAACTTTTCACCAAAGA
ACCCTTATGGCCCTCAACATCCACTATTACCCTTTATGTTACTGCTTCAACACTAGCC
CTTTCCATTGCTCTCCTTTTATGAACTCCCCTGCCTATACCAGATCGTCTAATTAATTT
TAATATAGGCCTCCTATTTATACTAGCAACATCAAGCCAGGCTGTCTACTCTATGAT
CAGAATGAGCATCTAATTCAAAATATGCACTGATCAGTGCATTACCAGCTGTGGCCC
AGACAATTTCATATAAGGTCACCCTAGCCATTATCCTGCTATCAGTTCTACTGATAA
GCAGCTCATTTAACTTATACACACTCATCACAATGCAAGAATTCCTCTGACTGCTCCT
ACCGTCATGGCCCCTAGCCATAATAT

>CHR 5 NT_034772.5 1804907-1804321
CATTTTTCTCTTCACTGTAGGTGGCCTAACCGGCATTGTATTAGCAAACTCATCATTA
GATATCATACTACACGATACATACTACGTCGTAGCTCACTTCCATTACGTTCTATCAA
TAGGAGCTGTATTCGCCATCATAGGGGGTTTTATCCACTGATTCCCTCTGTTCTCAGG
TTACACTCTAGACCAAACCTATGCCAAAATTCATTTTGCTATCATGTTCATTGGTGTA
AATCTAACTTTCTTCCCACAACACTTTCTTGGCCTATCTGGAATACCCCAACGTTACT
CGGACTACCCCGATGCATACACCACATGAAATATTCTATCATCTGTAGGCTCATTTA
TTTCCCTAACAGCAGTAATACTAATAATTTTTATAATTTGAGAAGGCTTCGCTTCAAA
ACGAAAAGCCCTAATAATCGAACAACCCTCCACCAACCTGGAGTGGCTATATGGAT
GCCTTCCACCCTATCATACGTTCGAAGAACCCATATACATAAAACCTAGACGAAAA
AGGAAGGAATGAAACCTCCTAAAGCTGGTTTCAAGCCAACCCCACAGCCTCTATGA
CTTTTTCAAAAAGA

>CHR 5 NT_034772.5 1803025-1802466
AACTATCTATAAACCTAGCCATGGCCATCCCCCTGTGAACAGGTACAGTAATTACAG
GCCTTCGCTCTAAAACCAAAAATGCCCTAGCCCATCTCCTACCACAGGGCACACCTG
CACCCCTTATTCCCATATTAATCATTATTGAAACCATCAGCCTACTTATTCAACCAGT
AGCCCTAGCCGTACGCCTAACTGCTAACATCACTGCAGGCCACCTACTTATGCACTT
AATTGGAAGTGCCACACTAGCAATATCAACTGTTAACCTCCCCTCAACGCTCATCAT
CTTCATAATCCTAATTCTATTAACTATTCTAGAAATCGCTGTTGCCCTAATCCAAGCC
TACGTTTTTACACTTCTAGTGAGCCCCTACCTACACGACAACACATAATGGCCCACC
AATCACATGCCTACCATATAGTAAAACCCAGCCCATGACCCCTAACGGGGGCCCTCT
CAGCCCTCCTGATAACCTCTGGCTTAGCCATATGATTCCACTTCTATTCCACAACCCT
ACTCACACTAGGCCTACTAACCAACGCGCTAACCATATACCAAT

>CHR 5 NT_034772.5 1802573-1801994
CCCTCTCAGCCCTCCTGATAACCTCTGGCTTAGCCATATGATTCCACTTCTATTCCAC
AACCCTACTCACACTAGGCCTACTAACCAACGCGCTAACCATATACCAATGGTGACG
TGATGTAATGCGAGAAAGCACGTACCAAGGCCACCACACACCACCCATCCAAAAAG
GTCTCCGATATGGAATAGTCCAATTTATCGCCTCAGAAATCTTCTTCTTCGCCAGATT
TTTCTGAGCTTTCTACCATTCCAGCCTAGCCCCTACCCCCCAACTAGGAGGACACTG
ACCTCCAACAGGCATCACCCCACTGAACCCCCAGAAGTCCCACTTCTGAACACATCC
GTACTACTCGCATCAGGAGTCTCAATCACTTGAGCCCACCACAGCCTAATAGAAAAT
AATCGAAACCAAATAATTCAAGCGTTACTTATTACAATTTTACTAGGTGTTTACTTTA
CCCTCCTACAAGCCTCAGAGTATTTCGAAACGCCCTTTACCATCTCTGACGACGGCA

TCTATGGCTCAACATTTTTTGTAGCCACAGGCTTTCACGGACTCCACGTCATCATTGG
ATCAACT

>CHR 5 NT_034772.5 1802112-1801539
TCCTACAAGCCTCAGAGTATTTCGAAACGCCCTTTACCATCTCTGACGACGGCATCT
ATGGCTCAACATTTTTTGTAGCCACAGGCTTTCACGGACTCCACGTCATCATTGGATC
AACTTTCCTCACTATTTGCCTCATCCGCCAACTAACATTTCACTTTACATCTAAACAT
CACTTCGGCTTTGAAGCCGCCGCCTGGTACTGACACTTCATAGATGTAGTCTGACTA
TTTCTATACGTCTCAATCTACTGATGAGGATCCTACTCTTTTAGTATAAACAGTACCG
TTAACTTCCAATTAACTAGTTTTGATGATATTCAAAAAAGAGTAATAAACTTCGCCC
TAATTCTAATAGTCAATACCCTCCTAGCCCTACTATTAATAGTTATTACATTCTGATT
ACCACAACTCAACAGTTACATAGAAAAATCCAACCCTTACGAATGTGGCTTCGACCC
CCTATTCCCCACCCGCATTCCTTTCTCCATGAAATTCTTCTTAGTAGCCATCACCTTT
CTATTATTTGACCTAGAAATTGCTCTCCTACTGCCCTTACCATGAGCCCTACAAAC

>CHR 5 NT_034772.5 1801633-1801071
CCTTTCTCCATGAAATTCTTCTTAGTAGCCATCACCTTTCTATTATTTGACCTAGAAA
TTGCTCTCCTACTGCCCTTACCATGAGCCCTACAAACAACCAACTTACCACTAACAG
TCATATCATCCCTCTTATTAATCATTATCCTAACCCTAAGCCTAGCCTACGAATGATC
ACAAAAGGGATTAGACTGAGCCGAATTGGTACATAGTTTAAATAAAACGAATGATT
TCGACTGATTAAATTATGATAGTCATATTTACCAAATGCCCCTTATTTATATAAATAT
TATACTAGCATTTACTATCTCACTTCTAGGAATATTAGTATACCGCTTACACCTGATA
TCCTCCCTACTATGTCTAGAAGGAATAATATTGTCATTATTTATTATGGCTACTCTCA
TAACCCTTAACACCCACTCCCTCTTAGCCAACATCGTGCCTATCACCATATTAGTCTT
TGCTGCCTGCGAGGCAGCAGTAGGCCTAGCCCTACTAGTTTCAATCTCCAACACATA
CGGCTTGGACTACGCCCATAACCTAAACCTACTCCAATGCTAAAA

>CHR 5 NT_006713.14 30541827-30541277
AACTGGGATTAGATACCCCACTATGCTTAGCCCTAAACTTCAACAGTTAAATTAACA
AAACTGCTCGCCAGAACACTACGAGCCACAGCTTAAAACTCAAAGGACCTGGCGGT
GCTTCATATCCCTCTAGAGGAGCCTGTTCTGTAATCGATAAACCCCGATCAACCTCA
CCACCTCTTGCTCAGCCTATATACCGCCATCTTCAGCAAACCCTGACGAAGGCTGCA
AAGTAAGCGCAAGTACCCACGTAAAGATGTTAGGTCAAGGTGTAGCCCATGAGCTG
GCAAGAAATGGGCTACATTTTCTACTTCAGAAAACTACGATAACCCTTATGAAATTT
AAGGGTCGAAGGTGGATTTAGCAGTAAACTAAGAGTAGAGTGCGTAGTTGAACAGG
GCCCTGAAGCGCGTACACACCGCCCGTCACCCTCCTCAAGTATACTTCAAAGGACAT
TTAACTAAAACCCCTGCGCATTTATATAGAGGAGATAAGTCGTAACATGGTAAGTGT
ACTGGAAAGTGCACTTGGACGAACCAGAGTGTAGCTTAACA

>CHR 5 NT_034772.5 36678748-36678073
CCTACTCCAATGCTAAAACTAATTGTCCCAACAATTATATTACTACCACTGACATGA
CTCTCCAAAAAACACATAATTTGAATCAACACAACCACTCACAGCCTAATTATTAGC
ACCATCCCCCTACTATTTTTCAACCAAATCAACAACAACCTATTTAGCTGCTCCTTAT
CCTTCTCCTCCGACCCCCTAACGACCCCCCTCCTAATACTAACTACCTGACTCCTGCC
CCTCACAATCATGGCAAGCCAACGCCACTTATCCAATGAACCACTATCACGAAAAA
AACTCTACCTCTCTATACTAATCTCCCTACAAATCTCCTTAATTATGACATTCACAGC

CATAGAACTAATCATATTTATATCTTCTTCGAAACCACACTTATCCCCACCCTGGCT
ATCATCACCCGATGAGGCAACCAACCAGAACGCCTGAACGCAGGTACATACTTCCT
ATTCTACACCCTAGTAGGCTCCCTCCCCCTACTCATCGCACTAATCTACACCCACAA
CACCCTAGGCTCACTGAATATTCTATTACTCACTCTTACTGCCCAAGAACTATCAAA
CTCCTGAGCCAATAACTTAATATGGCTAGCGTACACAATAGCTTTTATAGTAAAAAT
ACCTCTTTACGGTCTCCACCTATGGCTCCCTAAAGCCCATGTCGAAG

>CHR 5 NT_034772.5 36678265-36677713
CCCCTACTCATCGCACTAATCTACACCCACAACACCCTAGGCTCACTGAATATTCTA
TTACTCACTCTTACTGCCCAAGAACTATCAAACTCCTGAGCCAATAACTTAATATGG
CTAGCGTACACAATAGCTTTTATAGTAAAAATACCTCTTTACGGTCTCCACCTATGG
CTCCCTAAAGCCCATGTCGAAGCCCCCATCGCTGGGTCAATAGTACTTGCTGCAGTA
CTCTTAAAATTAGGCGGCTATGGTATAATACGCCTCACACTCATTCTCAACCCCCTG
ACAAAACATATAGCCTACCCCTTCCTTGTACTATCCCTGTGAGGCATAATTATAACA
AGCTCCATCTGCCTGCGACAAACAGACCTAAAATCGCTCATTGCATACTCTTCAATC
AGCCACATAGCCCTCGTAGTAACAGCCATCCTCATCCAAACCCCCTGAAGCTTCACC
GGCGCAGTCATTCTCATAGTCGCCCACGGACTTACATCCTCATTACTATTCTGCCTAG
CAAACTCAAACTACGAACGTACCCACAGTCGCATCATAA

>CHR 5 NT_034772.5 36677848-36677294
GTAACAGCCATCCTCATCCAAACCCCCTGAAGCTTCACCGGCGCAGTCATTCTCATA
GTCGCCCACGGACTTACATCCTCATTACTATTCTGCCTAGCAAACTCAAACTACGAA
CGTACCCACAGTCGCATCATAATTCTCTCTCAAGGACTTCAAACCCTACTCCCACTA
ATAGCCTTTTGATGACTTCTAGCAAGCCTCACTAATCTCGCCTTACCCCCCACTATTA
ACCTACTAGGAGAACTCTCTGTGCTAGTAACCACATTCTCCTGATCAAATATCACTC
TCCTACTTACAGGACTCAACATACTAATCACAGCCCTATACTCCCTCTACATATTTAC
CACAACACAATGAGGCTCGCTCAGCCACCACATTAACAACATAAAACCCTCATTCA
CACGAGAAAACACTCTCATGTTCATACACCTATCCCCTATCCTCCTCCTATCCCTCAA
CCCCGATATCATTACCGGGTTTTCCTCCTGTAAATATAGTTTAACCAAAACATCAGA
TTGTGAATCTGACAACAGAGGCTCACGACCCCTTATTTAC

>CHR 5 NT_034772.5 1799998-1799633
TGGCAAGCCTCGCCAACCTCGCTTTGCCCCCCACCATTAACCTACTAGGAGAACTCT
TCGTACTAGTGACCTCATTCTCCTGATCAAACATCACCCTCCTACTCACAGGACTTA
ACATACTAATCACAGCCCTATACTCCCTCTATATATTTACCACAACACAATGAGGCT
CGCTCACACACCACATTAACAACATAAAACCCTCACTTACACGAGAAAACACTCTC
ATATTCATACACCTATCCCCCATCCTCCTCCTATCCCTTAACCCTGATATCATTACCG
GTTTTACCTCCTGTAAATATAGTTTAACCAAAACATCAGATTGTGAATCTGATAACA
GAGGCTCACAGCCCCTTATTTACC

>CHR 5 NT_034772.5 36677381-36676818
CATTACCGGGTTTTCCTCCTGTAAATATAGTTTAACCAAAACATCAGATTGTGAATCT
GACAACAGAGGCTCACGACCCCTTATTTACCGAGAAAGCGCATAAGAACTGCTAAC
TCATATCCCCATGTCTAACAACATGGCTTTCTCAACTTTTAAAGGATAACAGCCATC
CGTTGGTCTTAGGCCCCAAAAATTTTGGTGCAACTCCAAATAAAAGTAATAACCATG
TACGCTACTATACCCACCCTAGCCCTAACTTCCTTAATTCCCCCTATCCTTACCACCC

TCATTAACCCTAATAAAAAAAACTCATATCCCCATTAAGTAAAATCCATTATCGCAT
CCACCCTTGTTATTAGTCTCTTCCCCACAACAATATTCATATACCTAGACCAAGAAGT
CATTATCTCAAACTGACACTGGGCAACAACCCAAACAACCCAACTCTCCCTAAGCTT
CAAACTAGATTATTTCTCCATAATATTTATCCCTGTAGCACTGTTCGTTACGTGATCC
ATCATAGAATTCTCACTATGATATATAAGCTCAGACCCAAACATTAAT

>CHR 5 NT_034772.5 1799289-1798769
CAAACAATACAACTCTCGCTAAGCGTTAAACTAGACTATTTCTCCATAATATTAATC
CCTGTAGCACTATTCGTCACATGATCCATCATAGAATTCTCACTATGGTATATAAGCT
CAGACCCAAACATTAATCAATTTTTCAAGTATCTGCTTATTTTCCTAATCACCATACT
AATCCTAGTTACAGCTAACAACCTATTCCAACTCTTCATCGGCTGAGAGGGCGTAGG
GATTATATCCTTCTTACTCATTAGCTGGTGGTATGCTCGAACAGATGCCAACACAGC
AGCCGTCCAAGCAATCCTATACAACCATATTGGTGACATCAGCTTCATCCTAGCCCT
AGCATGATTTTTCCTACACTCCAACTCATGAGAACCACAACAAATATTCCTCTTAAG
TGCTAACCCCAACCTTATCCCACTACTAGGTTTCCTCCTAGCAGCAGCAGGAAAATC
AGCTCAACTAGGCCTTCACCCCTGACTCCCCTCAGCCATGGAAGGCCCTACCCCTGT
CTCAGC

>CHR 5 NT_034772.5 36676591-36676006
TACACTCCAACTCATGAGACCCACAACAAATAACCCTCCTAAGTGCTAATCCAAGCC
TCACCCCACTACTAGGCTTCCTCCTAGCAGCAGCAGGCAAATCAGCTCAATTAGGCC
TTCACCCCTGACTCCCCTCAGCCATAGAAGGCCCCACCCCTGTCTCAGCCCTACTCC
ACTCAAGCACTATAGTTGTAGCAGGAGTCTTCCTACTCATCCGCTTCCACCCCCTAG
CAGAGAATAACCCACTAATCCAAACTCTCACACTATGCCTAGGCGCTATCACCACTC
TGTTCGCAGCAGTCTGCGCCCTTACACAAAATGACATCAAAAAAATCGTGGCCTTCT
CCACTTCAAGCCAACTAGGACTTATAATAGTCACAATCGGCATCAACCAACCACACC
TAGCATTCCTGCACATCTGTACCCACGCCTTCTTCAAAGCCATACTATTTATATGCTC
CGGATCCATCATCCACAACCTCAACAATGAACAAGATATTCGAAAAATAGGAGGAC
TACTCAAAACCATACCTCTTACTTCAACCTCCCTCACCATTGGCAACCTAGCATTGG
CAGGAATACCCTTCCT

>CHR 5 NT_034772.5 1798931-1798346
TACACTCCAACTCATGAGAACCACAACAAATATTCCTCTTAAGTGCTAACCCCAACC
TTATCCCACTACTAGGTTTCCTCCTAGCAGCAGCAGGAAAATCAGCTCAACTAGGCC
TTCACCCCTGACTCCCCTCAGCCATGGAAGGCCCTACCCCTGTCTCAGCCCTACTCC
ACTCAAGCACCATAGTTGTAGCTGGGGTCTTTCTACTCATCCGCTTCCACCCCCTAGC
AGAAAATAGCCCGCCAACCCAAACCCTCACACTATGTTTAGGCGCTATAACTACCCT
ACTCGCAGCAGTCTGCGCCCTCACACAAAATGATATCAAAAAAATCGTGGCCTTCTC
TACTTCAAGCCAATTGGGACTCATAATAGTCACAATCGGCATCAACCAGCCATACCT
AGCATTCCTACACATCTGTACCCACGCCTTCTTCAAAGCCATGCTATTTGTATGCTCC
GGATCCATTATCCATAACCTCAACAATGAACAAGACATTCGAAAAATAGGAGGACT
ACTCAAAACTATACCCCTCACTTCAACCTCCCTCACCATTGGCAGCCTAGCACTTGC
AGGAATACCCTTTCT

>CHR 5 NT_023148.12 2217633-2218198
AATTAGGACTCATGGTAGTCACAATCGGCATCAACCAACCATACCTAGCATTCCTAC
ACATCTGTACCCATGCTTTCTTCAAAGCCATATTATTTATATGCTCCGGATCCATTAT
TCATAACCTCAACAATGAACAAGACATTCGAAAAATAGGAGGACTACTCAAAATTA
TACCCCTCACTTCAACCTCCCTCACCATTGGCAGCCTGGCACTTGCAGGAATGCCCT
TCCTCACGGGCTTCTATTCCATAGACCTCATCATCAAAACCGCAAATATATCATACA
CCAACGCCTGAGCCCTATCTATTACTCTCATCGCCACTTCCCTAACAAGCGCCAATA
GCACTCGAATAATTCTCCTCACCCTAACAGGCCAACCTCGTTTCCCAACCCTAACCA
ACATCAACGAAAACAACCCTACCCTGCTAAGCCCCATCAAACGCCTAACAATCGGA
AGCCTATTCGCAGGATTTCTCATCACCAGCAACATTTTCCCCACATCCATCCCCCAA
ATGACAATCCCACTTCACTTAAAACTCACAGCCCTAGGCATCACCTTCCTAGGA

>CHR 5 NT_023148.12 2217748-2218306
TCATAACCTCAACAATGAACAAGACATTCGAAAAATAGGAGGACTACTCAAAATTA
TACCCCTCACTTCAACCTCCCTCACCATTGGCAGCCTGGCACTTGCAGGAATGCCCT
TCCTCACGGGCTTCTATTCCATAGACCTCATCATCAAAACCGCAAATATATCATACA
CCAACGCCTGAGCCCTATCTATTACTCTCATCGCCACTTCCCTAACAAGCGCCAATA
GCACTCGAATAATTCTCCTCACCCTAACAGGCCAACCTCGTTTCCCAACCCTAACCA
ACATCAACGAAAACAACCCTACCCTGCTAAGCCCCATCAAACGCCTAACAATCGGA
AGCCTATTCGCAGGATTTCTCATCACCAGCAACATTTTCCCCACATCCATCCCCCAA
ATGACAATCCCACTTCACTTAAAACTCACAGCCCTAGGCATCACCTTCCTAGGACTT
CTGACAGCCCTAGACCTCAACTACTTAACCAACAAACTCAAAATAAAAAACCCACT
GTGTACATTTCACTTTTCCAACATACTCGGATTCTATCCCTGTATTATT

>CHR 5 NT_023148.12 2218266-2218823
CACCGCACAATCCCCCACCTAAGCCTTCCCATAAGCCAAAATTTATCCCTACTTTTAT
TAGACCTAACTTGACTAGAAAAACTAATACCTAAAACAACCTCACAGTACCAAACC
TCCGCCTCCATCATCACCTCAACTCAAAAAGGCATAATCAAACTTTACTTCCTCTTTC
TTCTTCCCACTCCTCCTAACCCTCCTCCTAATCACATAACCTGTTACCCCGAGCACTC
TCAATTACAATATATACACCAACAAACAAGGTTCAACCAGTAACTACCACCAATCA
ATGCCCGTAATCGTATAAAGCCCCCGCACCAATAGGATCCTCCCGAATCAATGCTGG
CCCCTCCCCTTCATAAATTATTCAACTTCCTACACTATTAAAATTCACCACAATCACC
ACCCCATCATATTTTTTTACCCATAACACTAACCCCACTTCTATTGCTAATCCCACTA
AAACACTTACCAAGACCTCAATCCCTGACCCCCATGCCTCAGGATACTCCTCAATAG
C

>CHR 5 NT_034772.5 36675175-36674964
TTTACCACAACCACCACCCCATCATACTCTTTCACCCATAACACTAATCCTACTTCCA
TCACCAACCCCACTAAAACACTTACCAAGACCTCAACCCCTGACCCCCATGCCTCAG
GATACTCCTCAATAGC

>CHR 5 NT_034772.5 1797474-1796796
CACTAACCCTACCTCCATCGCTAACCCCACTAATACACTTACCAAGACCTCAATCCC
TGACCCCCATGCCTCAGGGTACTCCTCAATAGCTATCGCCATAGTATACCCAAAAAC
GACCATTATACCCCCTAAATAAATTTTAAAAAACCATTAAACCTATATAACCTCCCC
CATAATTTAAAATAATAACACACCCAACCACACCACTAACAATCAACACTAAACCC

CCATAAATAGGAGAAGGCTTAGAAGAAAACCCCACAAACCCCATTACTAAACCCAC
ACTCCATAAAAATAAAGCATATGTCATTATTCTCGCACGGACTACAACCGTGACCAA
TGATATGAAAAAACATCATTGCATTTCAACTACAAGAACACTAATGACCCCAACAC
GCAAAATCAACCCGTTAATAAAATTAATCAACCACTCATTTATTGACCTTCCCACTC
CATCCAATATCTCCACATGATGGAACTTCGGCTCATTTCTTGGCGCCTGCCTAATCCT
CCAAATCATCACAGGACTATTCCTAGCCATACACTACTCACCAGACGCTTCAACTGC
CTTCTCATTGATCGCCCACATCACCCGGGACGTGAACTATGGCTGAATTATCCGCTA
CCTCCACGCTAACGGAGTCGCAATATTCTTCATCTGCCTCTTCCTACACATCGG

>CHR5 NT_034772.5 36675134-36674448
CACTAATCCTACTTCCATCACCAACCCCACTAAAACACTTACCAAGACCTCAACCCC
TGACCCCCATGCCTCAGGATACTCCTCAATAGCCACCGCTGTAGTATATCCAAAAAC
AACCATCATTCCCCCCAAATAAATTAAAAAAACCATTAAACCCATGTAACCTCCCCC
GTAATTCAGAATAATAACACACCCAATCACACCACTAACAATCAATACTAAACCCC
CATAAATAGGAGAAGGCTTAGAAGAAAACCCCACAAACCCCATTACTAAACCCACA
CTCAACAGAAATAAAGCATACATCATTATTCTTGCACGGGCTACAACCACGACCAAT
GATATGAAAAACCATCATTGTATTTCAACTACAAGAACACTAATGACCCCAATACGC
AAAATTAACCCCTTGATAAAATTAATTAACCGCTCATTTATCGACCTCCCCACCCCA
TCCAATATCTCCGCATGATGAAACTTCGGCTCACTTCTTGGTGCCTGCCTAATCCTCC
AAATCACCACAGGACTATTCCTAGCCATACACTACTCACCAGACGCTTCAACCGCCT
TCTCATCGATCGCCCATATCACCCGAGACGTAAATTATGGCTGAATCATCCGCTACC
TTCACGCCAATGGCGCCTCAATATTCTTCATCTGCCTCTTCCTACACATCGGCCGAGG
CCT

>CHR 5 NT_006713.14 30541398-30540846
CCTCAAGTATACTTCAAAGGACATTTAACTAAAACCCCTGCGCATTTATATAGAGGA
GATAAGTCGTAACATGGTAAGTGTACTGGAAAGTGCACTTGGACGAACCAGAGTGT
AGCTTAACATAAAGCACCCAACTTACACTTAGGAGATTTCAACTCAACTTGACCACT
CTGAGCTAAACCTAGCCCCAAACCCATTCCACCTTACTACCAAACAACCTTAACCAA
ACCATTTACTCAAATAAAGTATAGGCGATAGAAATTGTAAACCGGCGCAATAGATA
TAGTACCGCAAGGGAAAGATGAAAAATTATAACCAAGCATAATATAGCAAGGACTA
ACCCCTGTACCTTCTGCATAATGAATTAACTAGAAATAACTTTGCAAAGAGAACCAA
AGCTAAGACCCCCGAAACCAGACGAGCTACCTAAGAACAGCTAAAAGAGCATACCC
GTCTATGTAGCAAAATAGTGGGAAGATTCATAGGTAGAGGCGACAAACCTACCGAG
TCTGGTGATAGCTGGTTGTCCAAGATAGAATCTTAGTTCAACTTT

>CHR 5 NT_023148.12 2219715-2220144
TACACGAAATAGGATCAAACAATCCCTTAGGCATCCCCTCCCATTCCGACAAAATTA
CCTTCCATCCTTACTACACAACCAAAGACATCCTAGGCCTATTCCTTTTCCTCCTCAC
CCTATTAACACTAGTACTATTCTCACCAGACCTCCTGGGCGACCCAGACAACTACAC
CCTGACTAACCCCCTAAACACCCCACCCCACATCAAACCCGAATGATATTTCCTATT
CGCCTACGCAATTCTCCGATCCATTCCCAATAAACTAGGAGGCATTCTAGCCCTTCT
ACTATCCATCCTCATCCTAGCAGCAATTCCTATACTCCACATATCCAAACAACAAAG
CATAATATTTCACCCATTAAGCCAATTCCTATATTGATTCCTAGCCACAGACCTTCTT
ACCCTAACCTGAATTGGAGGACAACCAGT

>CHR 5 NT_006713.14 30540924-30540284
AAGATTCATAGGTAGAGGCGACAAACCTACCGAGTCTGGTGATAGCTGGTTGTCCA
AGATAGAATCTTAGTTCAACTTTAAATTTACCCACAGAACCCTCCAAATCCCCCTGT
AAATTTAACTGTTAGTCCAAAGAGGAACAGCTCTTTGGACACTAGGAAAAAACCTT
GTAAAGAGAGTAAAAAATTTAATACCCATAGTAGGCCTAAAAGCAGCCACCAATTA
AGAAAGCGTTCAAGCTCAACACCTATCATCCGAAAAATCCCAAACATATGACTGAA
CTCCTTGCACCCAATTGGACCAATCTATTACCTTATAGAAGAACTAACGTTGGTATA
AGTAACATGAAAGCATTCTCCTCCACATAAGCCTACATCAGACCAAAATATTAAACT
GACAATTAACAGCCTAATATCTACAACCAACCAACAAGCCATTATTACCCCCACTGT
TAACCCAACACAGGCATGCCCACAAGGAAAGGTTAAAAAAAGTAAAAGGAACTCG
GCAAACCTTACCCCACCTGTTTACCAAAAACATCACCTCTAGCATTATCAGTATTAG
AGGCACCCCTGCCCGGTGACATATGTTTAACGGCCGCGGTACCCTAACCGTGCAAA
GGTAGCATAATCACTTGTTCC

>CHR 5 NT_023148.12 2220042-2220364
TATCCAAACAACAAAGCATAATATTTCACCCATTAAGCCAATTCCTATATTGATTCC
TAGCCACAGACCTTCTTACCCTAACCTGAATTGGAGGACAACCAGTGAACTACCCCT
TCACTACCATCGGACAAGTAGCATCCGTACTATACTTCACGACAATTTTAATCCTCA
TACCAGCCACCTCCTCAATCGAAAATAAAATACTCAAATGGACCTGCCCCTGTAGTA
TAAACCAATACACCGGTCTTGTAAACTGGAAATGAAGACTTCCTTCCAAGGACAAAT
CAGAGAAAAAGTACTTGACTTCACCATCAGCACCCAAA

>CHR 7 NT_079592.1 56744603-56744909
TTGCCCATGACCCCTAACAGGCATTTCTCCCCTTGACCCTCTGGAAGTACTCCTCCCG
AATACATCTGTATTACTTGCATCAGGGGTTTCAATTACCTGAGCCCATCACAGCCTA
ATAGAAAATAATCGAAAACAAATAATTCAAGCACTACTTATCACAATTACCTTACGT
ATTTACTTCACCCTCCTACAAGTCTCAGAATACTCTGAGGCTCCCTTTGCTATTTCTG
ATGGAATTTATGGCTCAACATTCTTTACACCTACAGGCTTTCACGGACTTCACATCGT
TATGGGATCAACATTCCTCACTATCTG

>CHR 8 NT_023678.15 902509-901844
AGCACACCCGTCTATGTAGCAAAATAGTGGGAAGATTCATGAGTAGTGGTGACAAG
CCTACCAAGCCTGGTAACAGCTGATTGTTTTCAACTTTAAACTTACCCACAGACTTA
CTTAATCTCCCTGTAACTTTAAGTGTTAGTCTAAAGAGGGACAGCTCTTTAGACATT
AGGAAACAACTGTCCTACAGGGAATAAAAAATATCACCACCATAGTTGGCATATAT
GGCCCAAAAGCAGCCACCAATTAAGAAAGCGTTCAAGCTCAACATCTAATTATCTA
AAATTCTAATCATCCTACTGAACTCCTAACATCACATTGGACTAATCTATTACTTAAT
AGAACCAGTAATGTTAATATAAGTAACATGAAGATATTCTCCATTGCATAAGCTTAT
ATCAGACCCCAATAATCCACTGACAGTTAACAGCCTAATATTAATAAGCAATATAAT
AAACACCCTATTATTTATACTGTTAATCCAACACAGGTATGCTCTAAGGAAAGATTA
CAAAAACTAAAAGGAACTCTGCAAGTCTTACCCTGCCTGTTTACCAAAAACATCACC
TCTAGCATTACGAGTATTAGAGGCACTGCCTGCCCAGTGACATATGTTCAATGGCCG
CAGTATCCTGACAGTGCAAAGGTAGCATAATCACTTGTTCC

>CHR 9 NT_008470.17 2194293-2194999
TTCCCTACTAGTCTCAATCTCCAACGCATAAGGCCTAGACTATGTACAAAATCTAAA
TTTACTCCAAKGCCAAAAATTATTATTCCAACAATTATACTGTTACAATATAACTCTC
CAAAAACTCTATAGTCTGAATATAGCTACTCATAGCCTATTCATCAGCCTCATTACC
CTATTATTTTTAATCAACCAAACAATAATTCATCTAACTTCTCATTAACTTTTTCTTC
TGACGCATTAACATCACCTCTTCTAATCCAAACAGCCTGACTACTACCTCTTATAATC
CTAGCAGGCCAACATCACCTATCTAATGAATCACCCTTATGGAAAAAAACTCCGTAT
TTCCAAATTAATTTTCCTACAACTTTTTTAAATTACAGCATTTACAGCCACAGAACTA
GTCATACTTTACATTCTTTTCGAAGCTACACTCATCCCTACCCTAATCATTATTACCT
GCTGAGGTAACCAACCAGAATGCCTAATGCAAGCTCATACTTATTTTACACACTAGT
AGGATCCCTTCCCTTACTTATTACACTCATCCACACCTGAAATAGCTCAGGTTCACTA
AATATCCTAATAATAATGTTTACTAATCAAGAACTATTAAACTTCTGATCTAACAAC
CTTATATGACTAGCATGTATTATGGCTTTTATAGTAAAAATACCCCTATACGGACTTC
ACCTATGACTCCCTAAAGCCCATGTCGAAGAAGCCGAATTCTGCAGATATCCATCAC
ACTGGCGGCCGCTCGAGCATGCATCTAGAGGGCCCAATTGCCCTATAGTAGTCGTT

>CHR 11 NT_009237.17 9317709-9317035
AGAGCACACCCGTCTATGTAGCAAAATAGTGGGAAGATTCGTAGGTAGAGGCGACA
AACCTACCGAGTCTGGTGATAGCTGGTTGTCCAAGACAGAATCTTAGTTCAACTTTA
AATTTACCCACAGAACCCTCCAAATCCCCCTGTAAATTTAACTGTTAGTCCAAAGAG
GAACAGCTCTTTGGACACTAGGAAAAAACCTTGTAAAGAGAGTAAAAAATTTAATA
CCCACAGTAGGCCTAAAAGCAGCCACCAATTAAGAAAGCGTTCAAGCTCGACACCT
GTCATCTAAAAAATCCCAAACACATAACTGAACTCCTTGCACCCAATTGGACCAATC
TATTACCCTATAGAAGAGCTAATGTTAGTATAAGTAACATGAAAACATTCTCCTCCG
CATAAGCCTACATCAGACCAAAATATTAAACTGACAATTAACAGCCCAATATCTATA
ACCAACCAACAAGCCATTATTACCCCCACTGTTAACCCAACACAGGCATGCCCACA
AGGAAAGGTTAAAAAAAGTAAAAGGAACTCGGCAAATCTTACCCCGCCTGTTTACC
AAAAACATCACCTCTAGCATTCTCAGTATTAGAGGCACCGCCTGCCCAGTGACATGC
GTTTAACGGCCGCGGTACCCTAACTGTGCAAAGGTAGCATAATCACTTGTTCC

>CHR 11 NT_009237.17 9318994-9318400
AGGTTTGGTCCTAGCCTTTCTATTAGCTCTTAGTAGGATTACACATGCAAGCATCCCC
GCCCCAGTGAGTCACCCTCTAAATCACCACGATCAAAAGGAACAGGTATCAAGCAC
GCAGCAACGCAGCTCAAAACGCTTAGCCTAGCCACACCTCCACGGGAGACAGCAGT
GATAAACCTTTAGCAATAAACCAAAGTTTAACTAAGCCATACTAACCCCAGGGTTGG
TCAATTTCGTGCCAGCCACCGCGGTCACACGATTAACCCAAGTCAATAGAAACCGG
CATAAAGAGTGTTTTAGATCAATTCCCCTCAATAAAGCTAAAATTCACGTGAGTTGT
AAAAAACTCCAGTTGATACAAAATAAACTACGAAAGTGGCTTTAATGCATCTGAAC
ACAGAATAACTAAGACCCAAACTGGGATTAGATACCCCACTATGCTTAGCCCTAAA
CTTCAACAGTTAAATTAACAAAACTGCTCGCCAGAACACTACGAGCCACAGCTTAA
AACTCAAAGGACCTGGCGGTGCTTCATATCCCTCTAGAGGAGCCTGTTCTGTAATCG
ATAAACCCCGATCAACCTCACCACCTCTTG

>CHR 11 NT_009237.17 9318585-9318008
GACCCAAACTGGGATTAGATACCCCACTATGCTTAGCCCTAAACTTCAACAGTTAAA
TTAACAAAACTGCTCGCCAGAACACTACGAGCCACAGCTTAAAACTCAAAGGACCT

GGCGGTGCTTCATATCCCTCTAGAGGAGCCTGTTCTGTAATCGATAAACCCCGATCA
ACCTCACCACCTCTTGCTCAGCCTATATACCGCCACCTTCAGCAAACCCTGACGAAG
GCCGCAAAGTAAGCGCAAGTACCCACGTAAAGACGTTAGGTCAAGGTGTAGCCCAT
GAGGTGGCAAGAAATGGGCTACATTTTCTACTTCAGAAAACTACGATAACCCTGATG
AAATTTAAGGGTCGAAGGTGGATTTAGCAGTAAACTAAGAGTAGAGTGCTTAGTTG
AACAGGGCCCTGAAGCGCGTACACACCGCCCGTCACCCTCCTCAAGTATACTTCAAA
GGACACTTAACTAAAACCCCTACGCATTTATATAGAGGAGATAAGTCGTAACATGGT
AAGTGTACTGGAAAGTGCACTTGGACGAACCAGAGTGTAGCTTAACACAAAGCACC
CAACTTACACTT

>CHR 11 NT_009237.17 9318150-9317598
CCTCAAGTATACTTCAAAGGACACTTAACTAAAACCCCTACGCATTTATATAGAGGA
GATAAGTCGTAACATGGTAAGTGTACTGGAAAGTGCACTTGGACGAACCAGAGTGT
AGCTTAACACAAAGCACCCAACTTACACTTAGGAGATTTCAACTCAACTTGACCGCT
CTGAGCCAAACCTAGCCCCAAACCCATTCCACCTTACTACCGAACAGCTTTAACCAA
ATCATTTACCCAAATAAAGTATAGGCGATAGAAATTGTAAATCGGCGCAATAGATA
TAGTACCGCAAGGGAAAGATGAAAAATTGCAACCAAGCATAATACAGCAAGGACTA
ATCCCTGTACCTTCTGCATAATGAATTAACTAGAAATAACTTTGCAAAGAGAACCAA
AGCTAAGACCCCCGAAACCAGACGAGCTACCTAAGAACAGCTAAAAGAGCACACCC
GTCTATGTAGCAAAATAGTGGGAAGATTCGTAGGTAGAGGCGACAAACCTACCGAG
TCTGGTGATAGCTGGTTGTCCAAGACAGAATCTTAGTTCAACTTT

>CHR 16 NT_037887.4 3360917-3360343
TTCTTAACAATATGCCTATAATCAATCTTCTCCTACTTATTATTACCACTTTAATCGCT
ATAGTATTCCTTACACTCATTGAATGAAAACTCTTAGGCTACACACAATTACGCAAA
GGACCTAACATTGTAGGTCCCCATGGACTGCTTCAACCATTCGCTGATGCAATAAAA
CTTTTCGTCAAAGAACCCTTACGTCCCTCAGCGTCTACTATTACCTTTTATACTACTG
CTCCAACCCTAGCTGTTTTGATCGCTCTCCTCTTGTGAACTCCCCTCCGCATACCAGA
TGCTCTAATTAATTTTAATATAGGCCTCCTATTTATACTAGCCACATCAAGCCTAGCC
ATCTACTCTATCCTATGATCAGGATGAGCATCTAATTCAAATTACGCACTAATCGGC
ACATTACAAGCTGTAGCCCAGACAATTTCATATGAAGTCACTCTAGCCATTATCCTT
TTATCAGTTCTACTAATAAGTGGCTCATTTAACTTACATGCATTTATCACAACGCAAG
AATTCCTCTGACTGCTTCTACCATCATAACCCCTAGCCATAATATGATTTATCTCC

>CHR 17 NT_024862.13 336116-336858
GGCCGAGGGCTGTACTATGGCTTATTTCTCTACCTAGAAACCTGAAACATTGGCATC
ATTCTCCTACTCACAACCATAGCAACAGCCTTCATAGGCTAGGTACTCCCATGAGGC
CAGATATCCTTGAGGGGCCACGGTAATCACAAATCTACTGTCCGCCATTCCTTATGT
CGGAACAGACCTGGTGCAGTGGGTTTGAGGTGGTTATTCAGTAGACACACCCACTCT
CACACGATTCTTCACCTTCCACTTTATCCTACCTTTCATTATTACAGCCCTAGTAGTC
TTACACCTACTATTTCTCCATGAGACAGCATCAAATAACCCCTCAGGAATCTCCTCC
CACTCCAACAAAATTACCTTCCACCCCTACTACATGACCAAAGATATCCTAGGCCTA
TTTCTCCTCCTTCTCACCCTAATAACACTAGTACTGTTTTCACCCGACCTCCTAACTG
ATCCAGATAACTACACCCTAGCCAACCGCCTGAATACTCCACCCCATATTAAACCCG
AATGGTATTTTCTATCTGCCTACGCAATCTTATGATCCATCCCCAACAAACTAGGAG
GCGTACTGGCCCTCCTACTATCAATCCTCATCCTAGCAGCAATTCCCACACTTCACAT

GTCCAAACAACAAAGCATAATATTCTGTCCACTAAACCAATTCCTATATTGACTCCT
AATTACAGATCTCCTTATCCTCACATGGGTT

>CHR 17 NT_024862.13 336858-337463
TTCATCACCTTCGGACAAACAGCATTCGTAGTTTACTTTGCTACAATTCTAGCCCTGA
TACCGCTTGCCTCCCTACTCGAAAACAAAATACTCAAATGGACCTGCCCTTGTAGTA
TAAACCAATATATTGGTCTTGTAAACCAGAAATGGAGACTCTCTCCCAAGGACAACT
CAGAGAAAAAGTACTTGACTTCACCATCAGCACCCAAAGCTAAGATTCTAATTTAAA
CTACTCTCTGTATTCTCATGAGGGAGCCATTTTGGGTACAACCCAAGTATCGACCCA
CCCCACCATAATTTTATATATATCGTGAATTTCTGCTAGCCACCATGAATATTATATA
GTACTATAATTGCTTAACTGTACATAGTACATATCAAACGAAACTCAACAACAACTC
CTCCCCACGACGCTTACAAGCAAGGACTAGAATACCTTAACTAACGGTAACACATT
AAACCCACCCTCCAAAAGAAACTTCTCAACCACAAGCGTACCAAAAAACACTATAT
ATCCTTGAGTACATAGTACATCTAATCGCTCATGGTAGATAGCACATTCCAGTTAAA
CAAGTCCTCGTCAATACGGATATCCCCCTCAGATAG

>CHR 17 NT_024862.13 337395-338087
ATCGCTCATGGTAGATAGCACATTCCAGTTAAACAAGTCCTCGTCAATACGGATATC
CCCCTCAGATAGTGGTCCGTTGTTCACCATCCTCTGTGAAATCAATATCCTGCAGAA
GAGTGCTACTCTTCTTGATCCAGGTCCATAATACTTGGGGGTAGCTATAATGAACTG
TATCCGGCATCTTGTTCTTACCTCACAGCCATAAAGTCAAGACCAACCACACGTTCC
CCTTAAATAAGACATCACGATGGATCACGGGTCTATTACCCTATTAATCAGTCACGG
GAGCTCTCCATGCATTTGGTATTTTAATCTGGGGGGTGTGCACGCGATAGCATTGTG
AAACGCTGGCCCCAGAGCACCCTATGTCGCAGTGTCTGTCTTTGATTCCTGCCCCAT
CCCATTATTGATCACACCTACATTCAATATCCCAGGCGAGCATACCTATCACAAGGT
GTTAATTAATTAATGCTTGTAGGACATAACAATCAGTAAACACACCCACGGCCGCTT
TTCACATCAACTCTACAATAAAATTCTGCAAATCCCCTTCCCCCATCTTTGGCCTCAC
CAACCCAGGAAAAGACATCTTTGCCAAACCCCAAAAACAAAAAGCCTTAATCCAAC
TCAACCAGAGCCCAAATTTTTATCTTTAGGCTGTACGCATTTTTAACAGTCACCCCTC
AACTAACA

>CHR 17 NT_024862.13 339611-340281
GAGCACACCCGTCTATGTAGCAAAATAGTGGGAAGATTCATAGGTAGAGGTGACAA
GCCTACCGAGCCTGGTGATAGCTGGTTGTCCAAGATAGAATCTTAGTTCAACTTTAA
ATTTATCTACAGAACCAGCTAATGCCTTTGTAAATTTAACTGTTAGTCTAAAGAGGG
ACAGCTCTTTAGACACTAGGAAAAAACCTTGTAGAGAGAGTAAAAAATATAACTTC
CAAAGTTGGCCTAGAAGAAGCGATCAATTAAGAAGGCGTTCAAGCTCAACACCCAA
CCACCCAAAAAATTCCAAACACATAACTGAACTCCTCACATCACATTGGACCAATCT
ATCATTTCATAGAAGAAATAATGTTAGTATAAGTGACATGCAAACATTCTCCTCCGC
ATAAGCCTACATCAGACCAAAATACTTCACTGACAATTAACAGCCCAATATCTATAA
ATAATCAATGAAACCATTATTACCCACACTGTTAACCCAACACAGGCATGCTTACAA
GGAAAGGTTAAAAAAAGTAAAAGGAACTCAGCGAATCTTACCCCTCCTGTTTATCA
AAAACATCACCTCTAGCATTACCAGTACTAGAGGCACCACCTGCCCAGTGACATATG
TTTAATGGCTGCGGTACCCTAACCGTGCAAAGGTAGCATAATCACTTG

>CHR 17 NT_024862.13 340090-340749
CACTGTTAACCCAACACAGGCATGCTTACAAGGAAAGGTTAAAAAAAGTAAAAGGA
ACTCAGCGAATCTTACCCCTCCTGTTTATCAAAAACATCACCTCTAGCATTACCAGT
ACTAGAGGCACCACCTGCCCAGTGACATATGTTTAATGGCTGCGGTACCCTAACCGT
GTAAAGGTAGCATAATCACTTGTTCCTTAAATAGGGACTTGTCTGAATGGCTCCACG
AGGGTTCAGCTGTCTCTTACTTTCAACCAGTGAAATTGACCTGCCCGTGAAGAGGCG
GACATAATACAACAAGACGAGAAGACCCTAAGGAGCTTTAATTTATGAATGCAAAC
AAGACCAAATAGGCCCGCAGGCCCTAAACTACCAGACCTGCGTTAAACATTTCGGT
TGGGGCGACCTCGGAGTATAACCTAACCTCCGAGCAACATATGCTGAGACTATACC
AGTCAAGGCGAATATCCACATACAATTGACCCAATAATTTGACCAACGGAACAAGT
TACCCTAGTGATAACAGTACAATCCTATTCTAGGGTCCACATCGACAGTAGCATTAA
CGACCTCGATGTTGGATCAGGACATCCCAATGGTGCAGCCGCTATTAAAGGTTCGTT
TGTTCAACGATTAGAGTCCTATGTGATCTGAGTTCAGA

>CHR 17 NT_0248612.13 340743-341346
GTTCAGACCGGAGTAATCCAGGTTGGTTTCTATCTATTCTACATTTCTTCCAGTACGA
AAGGATAAGAGAAATGGGGCCCACTTCATAAAGTGCCCCTGCCCCATAGATGATAT
TATCTCAGCATTTTACTGTACCCACACCCACCCAAGAACAGGGTTTGTTAAGATGGC
AGACCCCAGCAATTGCATAAAATTTAAAACTTTACAACCAGAGGTTCAACCCCTCTT
CTTAACAACATGTCCATAACTAACCTTCTACTTCTTATTGTACCTATCCTAATCGCCA
TGGCATTCTTCATGCTAACTGAATGAAAAATCCTAGGCTACATGCAACTACGCAAAG
GGCCCAATATTGTAGGCCCCTATGGACTATTACAACCTTTTGCCGATGCCATAAAAC
TCTTTACCAAAGAATCCTTAAAACCCTCAACGTCAACTGTTACCCTCTACATCACTG
CCCCAACCTTAGCCCTCTCTATTGCCCTTCTACTATGAACTCCCCTCCCTATACCTAA
CCCCCTAGTTAATCTTAACTTAGGCCTTCTATTCATTCTGGCTACATCCAGCCTAGCT
GTCTACTCTATCCTATGATCAGGATGAGCAT

>CHR 17 NT_024862.13 341020-341543
TAATCGCCATGGCATTCTTCATGCTAACTGAATGAAAAATCCTAGGCTACATGCAAC
TACGCAAAGGGCCCAATATTGTAGGCCCCTATGGACTATTACAACCTTTTGCCGATG
CCATAAAACTCTTTACCAAAGAATCCTTAAAACCCTCAACGTCAACTGTTACCCTCT
ACATCACTGCCCCAACCTTAGCCCTCTCTATTGCCCTTCTACTATGAACTCCCCTCCC
TATACCTAACCCCCTAGTTAATCTTAACTTAGGCCTTCTATTCATTCTGGCTACATCC
AGCCTAGCTGTCTACTCTATCCTATGATCAGGATGAGCATCAAACTCAAACTATGCC
CTGATCGGCGCATTATGAGCAGTTGCCCAAACAATTTCATACGAAGTTACCCTAGCT
ATCATCCTATTATCGATCCTACTAATAAGTGGCCAATTCAATCTCTGCACCCTCATTA
GAACACAAGAACACCTCTGACTACTCCTAACATCATGACCCCTAGCTATAATATGAT
TTATTTCC

>CHR 17 NT_010718.15 19103293-19103865
GAATTCCTCTGACTGCTCCTACCATCATGCCGCCTAGTCATAATATAATTTATCTCCC
CACTAGCAGAAACTAACCGAGCCCCTTTTGACCTAACAGAAGGAGAGTCAGAGTTA
GTCTCAGGCTTCAACATTGAATATGCCACAGGTTCATTTGCCCTCTTCTTTATGGCAG
AATACATGAATATTATCTTAATAAGTGCCCTAACTACTACTGTTTTCCTAGGAGCACT
ACACACTATATATTCACCAGAACTCTATACCACAAATTTCATTATCAAGACCCTCGT
TCTAACCGCCCTGATTTTATGAATTCGAACAGCATACCCCTGACTGTGCTACAACCA

ACTCATATATCTCCTATGAAAAAACTTCCTACCACTTAAACTAGCATTCTGCATATGT
TATATGGCCTGTCCTAATTTCCAGCATTCCACCTCAAACATAGGAAATATGTCTGAC
AAAAGAATTACTTTGATAGAGTAAATAATAGAGGTTAAAATCCTCTTATTTCTAGAA
CTATAGGAATTGAACCTACCCCTGATAATCCAAAATTCTCCATGCCACCTATTACAC

>CHR 17 NT_024862.13 341489-342064
ACACCTCTGACTACTCCTAACATCATGACCCCTAGCTATAATATGATTTATTTCCACA
CTAGCAGAAACCAATCGAGCTGCTTTTGACCTTACTGAAGGAGAATCAGAACTAGTC
TCAGGCTTCAACATTGAATATGCTGCAGGCCCATTCGCCCTATTCTTTATAGCCGAAT
ACATAAATATCATTAAAATAAATGCCCTAACTGCCATAATCTTCCTAGGTACAATAT
ATAATATTCACCTACCAGAACTCTACACAATATGCTTCACCATTAAAACCCTATTCCT
AACCTCTCTATTTTTATGAATCCGAACAGCATACCCCCGATTCCACTACGATCAACT
CATGTATCTCTTATGAAAAAATTTCTTACCATTTACACTAGCACTACTTATATGATAC
ATCTCAATATCCGCTACAATTTCCAACATTCCCCCCTAAACTTAAGAAATATGTCTG
ACAAAAGAGTGACTTTGATAGAGTAAATAATAGAGGTTTGAATCCTCTTATTTCTAG
GACCATAGGAATTGAACCTATCCCTGAGAATCCAAAATTCTCCGTGCCACGTGTCAC
AC

>CHR 17 NT_024862.13 338011-338489
CTTAATCCAACTCAACCAGAGCCCAAATTTTTATCTTTAGGCTGTACGCATTTTTAAC
AGTCACCCCTCAACTAACACGTCTTTTTCCCTCTTAGCCTCACTTCACAGACTCTACA
AATTTAGTTATCCCTTTTTCAGCCATCCCATTCAACGCATGCCCGACCACTACTAAAC
CAACACCCTAAACTAACCCAACCCCAAAGGCAAGGCTTTCCCCCCTACCCATTGCTT
GTATGCCCATCCCCTAGCCTTGCTTACACTGTGCAATCACAGTCTATGTAGCTTAATG
ACCCAAAGCAAGACACTGAAAATATCTAGATGGGCCTACACTGCCCCATAGACAAA
TAGGTTTGGTCCTAGCCTTTCTATTAACTCTTAGTAAGATTACACAAGCAAGCATCG
CCACCCTGGTGAAATAACCCTCTAAATCACTACAATCAAAAGGAGTAAGTATCAAG
CACGCATTAGTGCAGCTCAAA

>CHR 17 NT_024862.13 343174-343794
TCATATAAATTTATGTTAAACGCAGACTAAGAGCCTTCAAAGCCCTTAGTAAGTTAA
TAATACTTAATTTCTGCAGCACCCAAGGACTGCAAAACCCTACTTTGCATCTACTGA
ACGCAAATCAGCCACTTTAATTAAGCTAAGCCCTTGCTAGATCAATGGGACTTAAAC
CCACAAAAATTTGGTTAACAGCTAAATACCTTAATCAACTGGCTTCAATCTACTTCT
CCCGCCGCAAGGGAAAAAAGGCGGGAGAAGCCCAGGCAGGATTGAAGCTGCTTCTT
TGAATTTGCAATTCAACATGAAAGGCACCTCAGGGCTGGTAAAAAGAGAGTTTTTA
GATTTACAGTCTAATGCTTTGCTCAGCCATCTTACCCACTAATGTTCGCCAACCGCTG
ACTATTCTCAACAAATCATAAAGATATCGGAACACTATATTTATTGTTTGGTGCATG
AGCTGGAGTCTTGGGCACAGCCTTAAGCCTCCTTATTCAGACTGAACTAGGTCAACC
TGGCAACCTTCTAGGTAACGACCACATCTACAACGTCATCGTCACAGCCCACACATT
CTTCATAATCTTTTTCATAGTAATGTCTATCATAATCGGAGGCTTTGGCAA

>CHR 20 NT_011362.9 20987219-20986564
CACTGTTAACCCAACACAGGCATGCTCTAAGGAAACATTACAAAAAAATAAAAGGA
ACTCAGCAAATCTTACCCCGCCTGTTTACCAAAAACATCACCTCTAGCATTACCAGT
ATTAGAGGCACTATCTGCCCAGTGACATATATTCAACAGCTGTGGTATCCTGACCGT

GCGAAGGTAGCATAATCACTTGTTCTCTAAATAGGGACTTGAATGAATGGCCACACG
AAGGTTCAGCTGTCTCTTACTTTCAACCAGTGAAATTGATCTATCCGTGAAGAGGCG
GATATAAACAAATAAGACGAGAAAACCTTATGGAGCTTTAATTCATCAATGCAAAT
AAAAACTCCAACAAGCCTACAGGCCCTAGCCTCCTATCCCTGCATTAAAAATTTTGG
TTGGGGTGACCTCGGAGCATAATTCAACCTCCTAACAACACCTAAGACCACACAAG
TCTAAGTGAGTTATTACACATCGACCCAATAATTTGATAAACGGAGTAAGTTACCCT
AGGGATAACAGCGCAATCCTATACTAGAGTCCATATCGACAATAGAGTTTACGACCT
CGATGTTGGATCAGGACATCCTAATGGTGTAGCCGCTATTAAGAGTTTGTTTGTTCA
ATGATTAAAGTCCTATGTGATCTCAGTTCAGACC

>CHR X NT_011630.14 2761822-2761281
TTCTCCTACTTATTATCCCTACTTTCATCGCTATAGCATTCCTTACACTCATCGAATG
AAAAATCTTAGGCTACATACAACTACGCAAAGGACCTAACATTGTAGGTCCCTACG
GGCTGCTTCAACCATTCGCTAACGCAATAAAACTTTTCACCAAAGAACCCTTACAGC
CCTCAACATCTACTATTACCCTTTATATTATTGCTCCAATCCTAGCCCTTCCTATCGA
TCTCCTCTTATGAACTCCCCTCCCTATACCAAATCCTCTAATTAATTTTAACATAGGC
CTCCTATTTATACTAGCCATATCAATCCTAGCCATTTATTCTATGATCAGGATGAGCA
TCTAATTCAAAATATGCACTAATTGGCGCATCACGAGCTGTGGCCCAGACAATTTCA
AATGAGGTCACCCTAGCCATTATCCTGTTATCAGTTTTACTGATAAGTGGTTCATTTA
ACTTATATGCATTCATCACAATGCAAGAACTCCTCTGACTGCTCCTACCATCATTACC
CCTAGCCATAATGTGATTTATCCCC
